# Methodology and experiences of rapid advice guideline development for children with COVID-19: responding to the COVID-19 outbreak quickly and efficiently

**DOI:** 10.1186/s12874-022-01545-5

**Published:** 2022-04-03

**Authors:** Qi Zhou, Qinyuan Li, Janne Estill, Qi Wang, Zijun Wang, Qianling Shi, Jingyi Zhang, Xiaobo Zhang, Joseph L. Mathew, Rosalind L. Smyth, Detty Nurdiati, Zhou Fu, Hongmei Xu, Xianlan Zheng, Xiaodong Zhao, Quan Lu, Hui Liu, Yangqin Xun, Weiguo Li, Shu Yang, Xixi Feng, Mengshu Wang, Junqiang Lei, Xiaoping Luo, Liqun Wu, Xiaoxia Lu, Myeong Soo Lee, Shunying Zhao, Edwin Shih-Yen Chan, Yuan Qian, Wenwei Tu, Xiaoyan Dong, Guobao Li, Ruiqiu Zhao, Zhihui He, Siya Zhao, Xiao Liu, Qiu Li, Kehu Yang, Zhengxiu Luo, Enmei Liu, Yaolong Chen

**Affiliations:** 1 Evidence-Based Medicine Center, School of Basic Medical Sciences, Lanzhou University, Lanzhou, 730000 China; 2 Lanzhou University Institute of Health Data Science, Lanzhou, China; 3 Department of Respiratory Medicine Children’s Hospital of Chongqing Medical University, National Clinical Research Center for Child Health and Disorders, Ministry of Education Key Laboratory of Child Development and Disorders, Chongqing Key Laboratory of Pediatrics, Chongqing, China; 4 Institute of Global Health, University of Geneva, Geneva, Switzerland; 5 Institute of Mathematical Statistics and Actuarial Science, University of Bern, Bern, Switzerland; 6 Department of Health Research Methods, Evidence and Impact, Faculty of Health Sciences, McMaster University, Hamilton, Canada; 7 McMaster Health Forum, McMaster University, Hamilton, Canada; 8 The First School of Clinical Medicine, Lanzhou University, Lanzhou, China; 9 School of Public Health, Lanzhou University, Lanzhou, China; 10 Children’s Hospital of Fudan University, National Children’s Medical Center, Shanghai, China; 11 Advanced Pediatrics Centre, PGIMER Chandigarh, Chandigarh, India; 12 UCL Great Ormond Street Institute of Child Health, London, UK; 13 Cochrane Indonesia, Faculty of Medicine, Public Health and Nursing, Universitas Gadjah Mada, Yogyakarta, Indonesia; 14 Department of Infection Diseases Children’s Hospital of Chongqing Medical University, National Clinical Research Center for Child Health and Disorders, Ministry of Education Key Laboratory of Child Development and Disorders, Chongqing Key Laboratory of Pediatrics, Chongqing, China; 15 Department of Nursing Children’s Hospital of Chongqing Medical University, National Clinical Research Center for Child Health and Disorders, Ministry of Education Key Laboratory of Child Development and Disorders, Chongqing Key Laboratory of Pediatrics, Chongqing, China; 16 Department of Pediatric Research Institute, Ministry of Education Key Laboratory of Child Development and Disorders, National Clinical Research Center for Child Health and Disorders, China International Science and Technology Cooperation Base of Child Development and Critical Disorders, Children’s Hospital of Chongqing Medical University, Chongqing, China; 17 Shanghai Children’s Hospital Affiliated To Shanghai Jiaotong University, Shanghai, China; 18 Digital Institute of Medicine, Chengdu University of Traditional Chinese Medicine, Chengdu, China; 19 Department of Public Health, Chengdu Medical College, Chengdu, China; 20 Department of Radiology, the First Hospital of Lanzhou University, Lanzhou, China; 21 Department of Pediatrics, Tongji Hospital, Tongji Medical College, Huazhong University of Science and Technology, Wuhan, China; 22 Shenzhen Health Development Research Center, Shenzhen, China; 23 Department of Respiratory Medicine, Wuhan Children’s Hospital, Tongji Medical College, Huazhong University of Science and Technology, Wuhan, China; 24 Clinical Medicine Division, Korea Institute of Oriental Medicine, Daejeon, South Korea; 25 Korean Convergence Medicine, University of Science and Technology, Daejeon, South Korea; 26 Tianjin University of Traditional Chinese Medicine, Tianjin, China; 27 Beijing Children’s Hospital, Beijing, China; 28 Centre for Quantitative Medicine, Office of Clinical Sciences, Duke-National University of Singapore Medical School, Singapore, Singapore; 29 Singapore Clinical Research Institute, Singapore, Singapore; 30 Beijing Key Laboratory of Etiology of Viral Diseases in Children, Capital Institute of Pediatrics, Beijing, China; 31 Department of Pediatrics & Adolescent Medicine, Li Ka Shing Faculty of Medicine, University of Hong Kong, Hong Kong, China; 32 Shanghai Children’s Hospital, Shanghai, China; 33 National Clinical Research Center for Infectious Disease, Shenzhen, China; 34 Shenzhen Third People’s Hospital, Shenzhen, China; 35 Chongqing Ninth People’s Hospital, Chongqing, China; 36 Department of Nephrology Children’s Hospital of Chongqing Medical University, National Clinical Research Center for Child Health and Disorders, Ministry of Education Key Laboratory of Child Development and Disorders, Chongqing Key Laboratory of Pediatrics, Chongqing, China; 37 Key Laboratory of Evidence Based Medicine & Knowledge Translation of Gansu Province, Lanzhou, China; 38 WHO Collaborating Centre for Guideline Implementation and Knowledge Translation, Lanzhou, China; 39 GIN Asia, Lanzhou, 730000 China; 40 Lanzhou GRADE Centre, Lanzhou, 730000 China; 41 Research Unit of Evidence-Based Evaluation and Guidelines, Chinese Academy of Medical Sciences (2021RU017), School of Basic Medical Sciences, Lanzhou University, Lanzhou, 730000 China

**Keywords:** Methodology, Rapid Advice Guidelines, COVID-19, Indirect evidence

## Abstract

**Background:**

Rapid Advice Guidelines (RAG) provide decision makers with guidance to respond to public health emergencies by developing evidence-based recommendations in a short period of time with a scientific and standardized approach. However, the experience from the development process of a RAG has so far not been systematically summarized. Therefore, our working group will take the experience of *the development of the RAG for children with COVID-19* as an example to systematically explore the methodology, advantages, and challenges in the development of the RAG. We shall propose suggestions and reflections for future research, in order to provide a more detailed reference for future development of RAGs.

**Result:**

The development of the RAG by a group of 67 researchers from 11 countries took 50 days from the official commencement of the work (January 28, 2020) to submission (March 17, 2020). A total of 21 meetings were held with a total duration of 48 h (average 2.3 h per meeting) and an average of 16.5 participants attending**.** Only two of the ten recommendations were fully supported by direct evidence for COVID-19, three recommendations were supported by indirect evidence only, and the proportion of COVID-19 studies among the body of evidence in the remaining five recommendations ranged between 10 and 83%. Six of the ten recommendations used COVID-19 preprints as evidence support, and up to 50% of the studies with direct evidence on COVID-19 were preprints.

**Conclusions:**

In order to respond to public health emergencies, the development of RAG also requires a clear and transparent formulation process, usually using a large amount of indirect and non-peer-reviewed evidence to support the formation of recommendations. Strict following of the WHO RAG handbook does not only enhance the transparency and clarity of the guideline, but also can speed up the guideline development process, thereby saving time and labor costs.

**Supplementary Information:**

The online version contains supplementary material available at 10.1186/s12874-022-01545-5.

## Background

According to the World Health Organization (WHO), standard clinical practice guidelines (hereafter referred to as "standard guidelines") must be developed clearly and transparently following a rigorous methodology to obtain unbiased and high-quality recommendations and minimize the influence of conflicts of interest. The development of standard guidelines however will require on average about six months to two years to complete [1, 2]. Due to time constraints and the enormous input needed, developing standard guidelines is not the best way to assist decision-makers to rapidly make evidence-based decisions in a short period of time in case of public health emergencies. Thus, in 2006, the WHO introduced the concept and methodology of rapid advice guideline (RAG), which is characterized by short, scientific and standardized way of development. RAG aims to respond to potential public health emergencies or urgencies [3]. RAG has been proven successful with applications in several infectious diseases such as H5N1, HIV, and Ebola [4–6].

Since December 2019, a novel infectious disease (COVID-19) caused by severe acute respiratory syndrome coronavirus 2 (SARS-CoV-2) infection has swept through the world, posing significant challenges to public health safety and healthcare delivery systems worldwide [7]. In such public health emergencies, guideline developers face great challenges in making urgent reasonable recommendations. Therefore, the development of a RAG is one of the effective ways to address this issue [1, 2, 8, 9]. On January 28, 2020, the development of the first international rapid advice guideline for management of children with COVID-19 (hereinafter referred to as the "RAG for children with COVID-19"), following the WHO RAG methodology and led by the National Clinical Research Center for Child Health and Disorders, was launched. RAG for children with COVID-19 was initiated by the contributions of the WHO Collaborating Center for Guideline Implementation and Knowledge Translation. It took three months from approving RAG to its publication [10, 11]. This guideline focuses on the management of children younger than 18 years infected with SARS-CoV-2, and covers screening, diagnosis, treatment, and patient education. The guideline has so far been translated into 20 languages and accepted into the Guidelines International Network library [12]. Based on the practical experience from the "RAG for children with COVID-19", the aim of this article is to summarize the methodological processes and experiences in the development of the RAG, and provides suggestions and reflections on the future development of RAGs.

### Assessment of the need for the RAG

The WHO suggests to consider five factors when deciding to develop a RAG rather than standard guideline, or to delay the development of guideline. If all these five aspects are met, the development of a RAG is considered necessary. Therefore, prior to the official launch of the RAG for children with COVID-19, the core members (CMs: Liu E, Smyth RL, Chen Y, Luo Z, Li W, Zhou Q and Ren L) of the RAG working group assessed the need and rationale for the development of the RAG for children with COVID-19 based on these five aspects suggested by the WHO [1]. Having found that all five criteria were met, they concluded that it was necessary to develop a RAG for children with COVID-19 (Table 1).

**Table 1:** Results of the assessment of the need for a guideline for children with COVID-19

WHO criteria for the need of a RAG	Explanation	Is this criterion met?	Reason
Whether the public health event is an emergency or dangerous situation	1). Emergencies may be classified as natural, technological, or conflict related and may be of sudden onset (e.g., earthquakes, tsunamis, chemical crises) or more gradual onset (e.g., deteriorating situations in armed conflict, progressive disease outbreaks, drought, or food insecurities) 2). Using the rapid risk assessment of acute public health events manual to assess ‘‘any outbreak or other rapidly evolving situation that may have negative consequences for human health and requires immediate assessment and action’’ [13].	Yes	On January 26, the WHO raised the global risk of the epidemic to "high risk" for the first time, with 2014 cases confirmed in 11 countries in the Americas, Oceania, Asia and Europe [14]
Whether the public health event is novel	1). A new public health event (e.g., emerging infectious disease) or an event encountered previously but causing problems in a different context 2). If the contingency is not the first of its kind and relevant high-quality guideline already exists, it can be adopted directly or adapted to quickly address the health concern. (e.g., Ebola outbreak in the Democratic Republic of the Congo again in 2019, but by this time high-quality Ebola guidelines have been published, so there is no need to develop rapid advice guidelines for Ebola [15, 16].)	Yes	On January 26, nucleic acid testing of the virus by Chinese researchers had revealed that the disease was caused by a novel coronavirus, that it was an outbreak of a new public health threat, and that there were no high-quality guidelines for children with this condition published at this time [17, 18]
Duration of the public health event	The purpose of the RAG is to provide urgently needed evidence-based recommendations that should be implemented within one to three months, and if an event is likely to last more than six months, then a standard guide may be the best approach	Yes	The global SARS outbreak lasted almost 8 months from the first case to the complete eradication, the MERS outbreak has lasted 5 years, and SARS-CoV-2 belongs to the same coronavirus genus as SARS-CoV and MERS-CoV. COVID-19 epidemic can also be expected to last for some time, so it is feasible to develop rapid advice guidelines within 1 to 3 months of the outbreak [19]
The need to urgently address the problem of uncertainty	In the event of a public health emergency, there is significant controversy among health care professionals about certain issues or aspects that need to be resolved in the short term. (e.g., the use of glucocorticoids in the treatment of COVID-19 disease faces considerable controversy. The WHO Expert Group on the Clinical Management of Novel Coronaviruses, J Kenneth Baillie et al. commented in *the Lancet* that clinical evidence does not support the use of glucocorticoids to treat lung injury caused by novel coronaviruses, and may even be harmful, while China's frontline antiviral experts suggest that glucocorticoids have some benefit [20, 21].)	Yes	After a systematic review of published guidelines, we found that most focused on the diagnosis, treatment and prevention of COVID-19 in adults, with little attention paid to special populations such as children. There is an urgent need for clinical guidance on antiviral treatment, clinical manifestations, diagnostic criteria and home management of children with mild or severe COVID-19 [22]
Whether it can be rapidly and widely implemented	Before developing RAGs, it is important to consider the various factors involved in their implementation, such as the breadth and acceptability of the target population, their integration into national health policy systems, and their rapid implementation	Yes	SARS-CoV-2 is highly contagious and the entire population is susceptible. The total number of children in the world stands at 2 billion, and children, as a vulnerable group, are of great concern [23, 24]. In addition, countries threatened by the epidemic have elevated the prevention and control of COVID-19 to the highest national priority and have promulgated various health policies to prevent and control the disease [25], so that COVID-19 guidelines can be widely implemented

### Selection of development manuals

There are currently more than 60 manuals for the development of standard guidelines, including manuals issued by the Institute of Medicine (IOM), Canadian Medical Association (CMA), Scottish Intercollegiate Guidelines Network (SIGN), World Health Organization (WHO), American College of Physicians (ACP), and National Institute for Health and Care Excellence (NICE) [1, 26–30]. It generally takes one to two years to develop a standard guideline by following the methodology of these manuals [1, 26]. In response to public health emergencies, we need manuals that can shorten the time of guideline development. The GIN-McMaster guideline development checklist and the WHO RAG development handbook can help guideline developers complete a guideline in a short time [3, 31–33]. We compared the two manuals and finally chose the WHO RAG handbook for the following reasons: 1) The WHO RAG handbook has been successfully applied in the development of the RAGs of childhood tuberculosis treatment and HIV prevention and treatment in children and adolescents [34–36]; while GIN-McMaster has not yet been implemented in the development of RAGs; 2) We have recruited experts from WHO, who can provide us with support for the development of a RAG, and experts from WHO also recommended us to use the WHO RAG handbook; and 3) Both the WHO RAG handbook and the GIN-McMaster guideline development checklist were co-developed by experts from McMaster University, and their development processes were likely similar.

### The methodology and experience from the development process

#### Formulation of the RAG Working Group

The RAG was initiated on January 28, 2020 by the National Clinical Research Center for Child Health and Disorders. On February 3, 2020, one of the CMs (Li W) sent invitation letters to selected experts in the relevant disciplines. Sixty-seven members from 11 countries were invited and indicated their willingness to participate in the development of the guideline. Three working groups were formed (see Additional file 1): (I) A Guideline Development Group (GDG), which comprised 39 panelists from various disciplines, including pediatricians, infectious disease physicians, pulmonologists, epidemiologists, clinical pharmacists, methodologists, nurse practitioners, health economists, general practitioners, legal experts and global health researchers. The main responsibilities of the GDG were to draft the proposal, identify clinical questions, reach consensus on the recommendations with use of a Delphi approach, and approve the final version of the guidelines; (II) A Rapid Review Group (RRG), which comprised 26 evidence synthesis methodologists and pediatricians. The primary responsibilities of the RRG were to collect clinical questions, develop rapid reviews, assess the quality of evidence, prepare recommendation decision forms and take meeting minutes; and (III) Patient Representatives (PR), which comprised of two guardians of children with COVID-19. We did not recruit children or adolescent themselves for the following reasons:1) at that time, no policy for recruitment of children with COVID-19 existed and the ethics committee of Children’s Hospital of Chongqing Medical University did not permit us to recruit children with COVID-19; 2) at the time of the development of the RAG, only few children had been diagnosed with COVID-19 and the guardians tended to be reluctant to involve their children in research [37, 38]; and 3) children, especially those under 6 years of age, are also not necessarily able to express themselves clearly enough [39]. The PRs were involved in the voting process for recommendations and providing feedback on the full text of the guideline.

### Declaration and management of conflicts of interest

To ensure clarity and transparency in the development of the RAG, all participants were required to declare any potential conflicts of interest that may be relevant to the RAG [40, 41]. One CM (Li W) collected the conflicts of interest declaration forms among all members participating in the development of the RAG through email. Participants were required to declare any financial, professional and other interests that may be related to the RAG. The declared conflict of interests was reviewed by the CMs (Liu E, Chen Y, Li W, Zhou Q). Based on the assessment of conflict of interests, we limited the participation of experts with potential conflict of interests in the core work or excluded them from the overall development of the guideline. All disclosures and conflicts of interest management decisions have been publicly reported [42, 43].

### Registration of the guideline and publication of the protocol

The RAG was registered on the International Practice Guidelines Registry Platform (registration No. IPGRP-2020CN008). The protocol was published on March 26 of 2020 [11].

### Collection and prioritization of clinical questions

Four CMs (Liu E, Chen Y, Li W, Zhou Q) conducted a scoping review of the published literatures related to COVID-19, and developed the initial clinical questions related to children with COVID-19 [44]. After two rounds of discussions among three CMs (Liu E, Smyth RL, Li W) and clinical experts working in the frontline of infectious disease prevention, 26 clinical questions were identified for further assessment of their importance (Table 2).

**Table 2:** List of clinical questions

No	Initial Questions (IQ)	Average score	Decision of inclusion; reason, remarks	Final Questions
IQ1	What are the main symptoms of children infected with the novel coronavirus?	6.33	**Yes.** According to experts’ comments, we revised the wording of this clinical question	**Clinical question 1:** What are the symptoms of children with COVID-19 and who needs further assessment?
IQ2	What is confirmatory test for diagnosis novel coronavirus infection in children?	6.19	**Yes.** According to experts’ comments, we combined IQ2 and IQ8 into one question	**Clinical question 3:** Should computed tomography (CT) scan be used for the diagnosis and monitoring of children with COVID-19?
IQ3	How to conduct screening to novel coronavirus for suspected childhood infection in hospitals?	6.17	**No.** According to experts’ comments, this clinical question was not a priority in this phase	NA
IQ4	How to hierarchically manage children with novel coronavirus infection (including asymptomatic infected children)?	5.96	**Yes.** According to experts’ comments, the clinical question is answered in the form of a treatment pathway diagram	For the pathway diagram, please refer to the original maintext of the rapid advice guidelines for management of children with COVID-19 [10]
IQ5	How to effectively prevent children getting infected novel coronavirus? (Tips for protecting children from novel coronavirus infection)?	5.96	**No.** According to experts’ comments, this clinical question not belong to our guideline scope	NA
IQ6	Should new antiviral medications (such as lopinavir/ritonavir, remdesivir (GS-5734)) be used to treat in children with novel coronavirus infection?	5.85	**Yes.** According to experts’ comments, we combined IQ6 and IQ9 into one question and revised the wording	**Clinical question 4:** Should antiviral drugs such as ribavirin, interferon, remdesivir (GS-5734), lopinavir/ritonavir or oseltamivir be used to treat children with COVID-19?
IQ7	Should systemic corticosteroid be used to treat children with novel coronavirus infection?	5.83	**Yes.** According to experts’ comments, we revised the wording of this clinical question	**Clinical question 6:** Should systemic corticosteroids be used to treat children with severe COVID-19?
IQ8	Do children with non-severe novel coronavirus infection need imaging tests?	5.69	**Yes.** According to experts’ comments, we combined IQ2 and IQ8 into one question	**Clinical question 3:** Should computed tomography (CT) scan be used for the diagnosis and monitoring of children with COVID-19?
IQ9	Should traditional antiviral medications (such as ribavirin, interferon) be used to children with novel coronavirus infection?	5.63	**Yes.** According to experts’ comments, we combined IQ6 and IQ9 into one question	**Clinical question 4:** Should antiviral drugs such as ribavirin, interferon, remdesivir (GS-5734), lopinavir/ritonavir or oseltamivir be used to treat children with COVID-19?
IQ10	Where could parents and their children get reliable and evidence-based information about novel coronavirus epidemic and prevention?	5.63	**Yes.** According to experts’ comments, we revised the wording of this clinical question	**Clinical question 10:** How should parents be advised to get information on SARS-CoV-2 infection?
IQ11	How to manage a child who has history of epidemiological exposure but without symptoms?	5.57	**Yes.** According to experts’ comments, we combined IQ6 and IQ9 into one question	**Clinical question 2:** How should children who have had contact with COVID-19 patients be managed?
IQ12	Should IVIG be used to treat children with severe novel coronavirus severe infection?	5.56	**Yes.** According to experts’ comments, we revised the wording of this clinical question	**Clinical question 7:** Should intravenous immunoglobulin (IVIG) be used to treat children with severe COVID-19?
IQ13	For children with no history of epidemiological exposure, what are the indications or symptoms for screening novel coronavirus?	5.37	**No.** We reached consensus that this clinical question was not a priority issue for this guideline	NA
IQ14	Should CT test be better than normal chest X-ray in children with severe novel coronavirus pneumonia?	5.35	**No.** We reached consensus that this clinical question was not a priority issue for this guideline	NA
IQ15	What are the imaging features of children with novel coronavirus infection in lungs, are they specific?	5.35	**No.** We reached consensus that this clinical question was not a priority issue for this guideline	NA
IQ16	How long is the average incubation period for children infected with novel coronavirus?	5.31	**No.** We reached consensus that this clinical question was not a priority issue for this guideline	NA
IQ17	What is the severity for novel coronavirus infection in children compared to adults?	5.15	**No.** We reached consensus that this clinical question was not a priority issue for this guideline	NA
IQ18	What is the prognosis of children with novel coronavirus infection?	5.12	**No.** We reached consensus that this clinical question was not a priority issue for this guideline	NA
IQ19	How to conduct psychological assessment and therapy for children diagnosed with novel coronavirus infection?	4.96	**Yes.** We reached consensus that this clinical question was a priority issue for this guideline	**Clinical question 8:** What is appropriate supportive care for children with severe COVID-19?
IQ20	Could the novel coronavirus be vertically transmitted (mother to infant, breastfeeding)? If so, is there any difference in risk between natural delivery and caesarean section?	4.94	**Yes.** We reached consensus that this clinical question was a priority issue for this guideline	**Clinical question 9:** Should mothers with COVID-19 continue to breastfeed their babies?
IQ21	Should antibiotic agents be used to treat novel coronavirus infection?	4.91	**Yes.** According to experts’ comments, this clinical question was a priority issue for this guideline	**Clinical question 5:** Should antibiotics be used to treat children with COVID-19?
IQ22	What are the predisposing factors (gender, age, underlying diseases, ethnic differences) for novel coronavirus infection in children?	4.85	**No.** According to experts’ comments, this clinical question was not a priority issue for this guideline	NA
IQ23	Does the complete blood count (CBC) test for children with early 2019-nCoV infection have a predictive effect on the severity of the disease?	4.85	**No.** According to experts’ comments, this clinical question was not a priority issue for this guideline	NA
IQ24	Could a multidisciplinary cooperation improve the outcomes for children with severe novel coronavirus infection?	4.62	**No.** We reached consensus that this clinical question was not a priority issue for this guideline	NA
IQ25	How susceptible are children compared to adults for novel coronavirus?	4.33	**No.** We reached consensus that this clinical question was not a priority issue for this guideline	NA
IQ26	What is the severity for 2019-nCoV infection in children (e.g., mortality and ICU admission rates) compared to SARS/MERS?	4.28	**No.** We members reached consensus that this clinical question was not a priority issue for this guideline	NA

*IQ* Initial Question, *NA* Not Applicable

On February 2, 2020, one CM (Li W) sent a clinical questionnaire to 33 GDG members by email. All experts scored each question from 1 to 7 according to its importance (1: the question is not important at all and should not be included in the guideline; 7: the question is extremely important and must be included in the guideline). Twenty-seven (81.8%) of the invited 33 GDG members completed the questionnaire. On February 7, 2020, two CMs (Li W, Zhou Q) prioritized the clinical questions according to the questionnaire scores and modified the questions based on the feedback from the GDG. After considering the score ranking and discussing the feedback, six CMs (Liu E, Smyth RL, Chen Y, Luo Z, Li W, Zhou Q) reached an agreement and included ten clinical questions (Table 2).

### Evidence retrieval, assessment and synthesis

Based on the ten selected clinical questions, the RRG conducted 11 rapid reviews [45–55]. Each rapid review was assigned specific inclusion and exclusion criteria, based on the amount of available direct or indirect evidence on COVID-19 in children. In the event of sufficient available studies, we included only direct evidence from COVID-19. If there were no studies on COVID-19 in children or indirect evidence from adults, we also included indirect evidence from other coronavirus respiratory infections such as Severe Acute Respiratory Syndrome (SARS) or Middle East Respiratory Syndrome (MERS) to answer the clinical questions.

The RRG systematically searched the following databases: Cochrane library, MEDLINE (via PubMed), Embase, Web of Science, CBM (China Biology Medicine), CNKI (China National Knowledge Infrastructure) and Wanfang Data. The search terms were COVID-19, 2019-CoV, Novel coronavirus, 2019-nCoV, Middle East Respiratory Syndrome Coronavirus, MERS, MERS-CoV, Severe Acute Respiratory Syndrome, SARS, SARS-CoV, and their synonyms, as well as terms related to specific the topic of each review. We developed the search strategy in cooperation with an experienced medical information retrieval specialist [5, 56]. The first searches were completed on 28 February 2020. Later, due to the length of the development process of the RAG, the searches were updated on March 31, 2020. In addition, the RRG also manually searched SSRN (https://www.ssrn.com/index.cfm/en/), medRxiv (https://www.medrxiv.org/) and bioRxiv (https://www.biorxiv.org/) preprint platforms and Google Scholar, and tracked references of the included studies.

Since another high-quality rapid review on the same topic was published, the rapid review on quarantine to control COVID-19 was discontinued on 8 April 2020 [45, 57]. Finally, the RRG completed 13 rapid reviews (see Additional file 2). The GRADE method was used to grade the evidence from the results of the rapid reviews [45, 58]. The detailed grading results were reported in the published articles. The questions and supporting evidence from the rapid reviews is shown in Table 3.

**Table 3:** The questions and supporting evidence from rapid reviews

Questions	Rapid review(s) to answer the questions	Studies included in the rapid review	Was the recommendation fully supported by evidence from COVID-19 (Yes/Partially/No)	Was the recommendation fully supported by evidence from children with COVID-19 (Yes/Partially /No)	Proportion of preprints in the COVID-19 studies per rapid review
**Clinical question 1:** what are the symptoms of children with COVID-19 and who needs further assessment?	One rapid review (produced by RRG)	Wang Z et al., 2020 [45] **Number of primary studies:** 49 **Study design:** 25 case reports, 23 case series and one cohort study **Patients:** All patients are children with COVID-19 **Sources:** Seven preprints, 42 journal articles	Yes, 100%	Yes, 100%	14.3% (7/49)
**Clinical question 2:** how should children who have had contact with COVID-19 patients be managed?	Three rapid reviews(two rapid reviews produced by RRG, one published article)	Gao Y et al., 2020 [46] **Number of primary studies:**9 **Study design:** 9 cross-sectional studies **Patients:** One article was about COVID-19 (all patients were adults), eight articles were about SARS **Sources:** Zero preprint, 9 journal articles	Partially, 11%	No, 0%	0.0%(0/4)
		Zhou Q et al., 2020 [47] **Number of primary studies:** 40 **Study design:** Nine case series, 30 cross-sectional studies and one case–control **Patients:** Four articles were about adults with COVID-19, 25 articles were about SARS, 11 articles were about MERS **Sources:** Zero preprint, 40 journal articles	Partially, 10%	No, 0%	0.0%(0/1)
		Nussbaumer-Streit B et al., 2020 [57] **Number of primary studies:**29 **Study design:** Four cohort studies and 25 modelling studies **Patients:** 10 articles were about COVID-19 (almost patients were adults), 15 articles were about SARS, two articles were about MERS and two articles were about SARS and other infectious diseases **Sources:** Zero preprint, 28 journal articles and one unpublished report	Partially, 34%	No, 0%*	0.0%(0/10)
**Clinical question 3:** should computed tomography (CT) scan be used for the diagnosis and monitoring of children with COVID-19?	One rapid review (produced by RRG)	Lv M et al., 2020 [48] **Number of primary studies:** 103 **Study design:** 82 case series and 21 case reports **Patients:** All articles were about COVID-19, 7 articles were only included children, other articles were included adults **Sources:** Five preprints, 98 journal articles	Partially, 100%	Partial, 6.8%^a^	4.8%(5/103)
**Clinical question 4:** should antiviral drugs such as ribavirin, interferon, remdesivir (GS-5734), lopinavir/ritonavir or oseltamivir be used to treat children with COVID-19?	One rapid review (produced by RRG)	Shi Q et al., 2020 [49] **Number of primary studies:** 23 **Study design:** Six randomized controlled trials and 17 cohort studies **Patients:** Seven articles were about COVID-19 (almost patients were adults), 13 articles were about SARS, three articles were about MERS **Sources:** Three preprints, 20 journal articles	Partially, 30%	No, 0%^a^	42.9%(3/7)
**Clinical question 5:** should antibiotics be used to treat children with COVID-19?	One rapid review (produced by RRG)	Wang J et al., 2020 [50] **Number of primary studies:** Six **Study design:** Five case series and one cohort study **Patients:** Five articles were about SARS and one article was about MERS **Sources:** Zero preprint, six journal articles	No, 0%	No, 0%	NA
**Clinical question 6:** should systemic corticosteroids be used to treat children with severe COVID-19?	One rapid review (produced by RRG)	Lu S et al., 2020 [51] **Number of primary studies:** 23 **Study design:** One randomized controlled trial and 22 cohort studies **Patients:** Five articles were about COVID-19 (all patients were adults), 17 articles were about SARS and one article was about MERS **Sources:** Two preprints, 21 journal articles	Partially, 22%	No, 0%	40.0% (2/5)
**Clinical question 7:** should intravenous immunoglobulin (IVIG) be used to treat children with severe COVID-19?	One rapid review (produced by RRG)	Zhang J et al., 2020 [52] **Number of primary studies:** Six **Study design:** One randomized controlled trial, four case series and one case report **Patients:** Two articles were about COVID-19 (all patients were adults) and four articles were about SARS **Sources:** One preprint, five journal articles	Partially, 33%	No, 0%	50.0% (1/2)
**Clinical question 8:** what is appropriate supportive care for children with severe COVID-19?	One umbrella review (produced by RRG)	Luo X et al., 2020 [53] **Number of primary studies:** 18 **Study design:** 18 systematic reviews **Patients:** 18 articles were not about COVID-19, SARS and MERS **Sources:** Zero preprint, 18 journal articles	No, 0%	No, 0%	NA
**Clinical question 9:** should mothers with COVID-19 continue to breastfeed their babies?	Two rapid reviews (one rapid review produced by RRG, one previously published article)	Yang N et al., 2020 [54] **Number of primary studies:** Six **Study design:** Five case reports and one case series **Patients:** Five articles were about COVID-19 (all patients were mothers) and one article was influenza **Sources:** Zero preprint, six journal articles	Partially, 83%	No, 0%	0.0% (0/5)
		Jefferson T et al., 2011 [59] **Number of primary studies:** 67 **Study design:** Six randomized controlled trials, 17 non-randomized controlled trials, nine case–control studies, 22 cohort studies and 13 before-after controlled studies **Patients:** 67 articles were not about COVID-19 and MERS **Sources:** Six journal articles	No, 0%	No, 0%	NA
**Clinical question 10:** how should parents be advised to get information on SARS-CoV-2 infection?	One rapid review (produced by RRG)	Li W et al., 2020 [55] **Number of primary studies:** 24 **Study design:** 24 cross-sectional studies **Patients:** Six articles were about COVID-19 (almost patients were adults), eight articles were about SARS, ten articles were about MERS **Sources:** Zero preprint, 24 journal articles	Partially, 25%	No, 0%^a^	0.0% (0/6)

*RRG* Rapid Review Group

^a^Only a small population of the included COVID-19 studies were pediatric patients, so we do not consider evidence from these studies as evidence from children with COVID-19

### Formulation of recommendations

Based on the ten clinical questions and supporting evidence from rapid reviews, six CMs (Liu E, Smyth RL, Chen Y, Luo Z, Zhou Q, Li W) initially drafted ten recommendations. The relevant materials and questionnaires were sent to the GDG members by email in electronic format, and also to the two children’s guardians in paper versions. The preferences and values of the two children’s guardians and clinical experts were consistent. Two rounds of Delphi survey were conducted on February 24, 2020 and February 28, 2020, respectively, and we voted on the recommendations. The consensus criterion was that at least 70% of the invited panelists agreeing on the recommendation [45, 60]. After each round of Delphi survey, the core group discussed the feedback, revised the recommendations and also disclosed the feedback to the panel members before the next round. Detailed expert feedback can be found in Additional file 3.

A total of nine recommendations reached consensus in the first round of Delphi survey, leaving only one recommendation to be determined (*Recommendation #3: CT scan should not be used routinely in the diagnosis of COVID-19 in children, although it may be helpful in monitoring children who develop severe respiratory symptoms*). Six CMs (Liu E, Smyth RL, Luo Z, Chen Y, Zhou Q, Li W) revised the wording and rationale of the recommendations based on the first round of Delphi survey. Afterwards, a second round of Delphi survey was conducted for two recommendations: recommendation #3 for which consensus was not reached in the first round, and recommendation #6 (*Recommendation #6: systemic glucocorticoids should not be used routinely for children with COVID-19. Only low-dose and short-duration systemic glucocorticoid therapy can be used for children with severe COVID-19 in the context of clinical trials*). The recommendation was divided into two sub-recommendations (*a. We recommend against using systemic glucocorticoids for children with COVID-19 routinely; b. We suggest a low dose and a short duration for severe COVID-19 children only when over inflammatory reaction or in the context of clinical trials*) after feedback from the first round. Consensus were reached for both recommendations, resulting in a total of ten recommendations being finally included in the RAG. The panelists made a total of 181 comments during the two rounds of Delphi survey (Table 4).

**Table 4:** Results of the two rounds of Delphi survey

	**First Round Delphi Process (results from 33 panelists)**				**Second Round Delphi Process (results from 30 panelists)**			
	**Preliminary recommendation (1**^**st**^** round)**	**Level of agreement**	**How many panelists gave comments**	**Total number of comments**	**Preliminary recommendation (2**^**nd**^** round)**	**Level of Agreement**	**How many panelists gave comments**	**Total number of comments**
CQ1	The main symptoms of children with COVID-19 are fever and cough. The symptoms of COVID-19 are usually less severe in children than adults. Leukocyte and lymphocyte counts are usually normal. Chest imaging findings do not significantly differ between adults and children. (2C)	94% (Consensus)	10	11	The main symptoms of children with COVID-19 are fever and cough. The symptoms of COVID-19 are usually less severe in children than adults. Leukocyte and lymphocyte counts are usually normal. Although there is no significant difference between adults and children in chest imaging characteristics, the extend of the abnormalities are usually less in children. (2C)	NA	8	11
CQ2	We recommend that children who have been in close contact with COVID-19 patients are initially evaluated by their guardians or family doctors. If no obvious symptoms are found, we recommend staying at home for observation; if there are obvious symptoms such as fever and cough, we recommend further evaluation in the hospital. (2C)	94% (Consensus)	9	11	We suggest that children who have been in close contact with COVID-19 patients are initially evaluated by their guardians or family doctors. If no obvious symptoms occur, we recommend staying at home for observation for a duration of at least 14 days; if there are obvious symptoms, we suggest further evaluation in the hospital. (2C)	NA	9	12
CQ3	We recommend X-ray rather than CT to assist in diagnosis of COVID-19 in children if necessary. (2C)	55% (Not consensus)	13	16	We suggest not using imaging test as routine examination for children with COVID-19. (2C)	79% (Consensus)	11	13
CQ4	We recommend against using antiviral drugs for children with COVID-19. Specific antiviral drugs may be administered only in the context of clinical trial (1C)	94% (Consensus)	7	9	We recommend against using antiviral drugs for children with COVID-19. Specific antiviral drugs may be administered only in the context of clinical trial. (1C)	NA	4	7
CQ5	We recommend against the use of antibiotic agents for children with COVID-19 when there is no evidence of bacterial coinfection. (1B)	100% (Consensus)	2	3	We recommend against using antibiotic agents for children with COVID-19 when there is no evidence of bacterial coinfection. (1B)	NA	1	1
CQ6	We recommend using systemic glucocorticoids with a low dose and for a short duration for children with severe COVID-19. (2C)	79% (Consensus)^a^	12	12	1) We recommend against using systemic glucocorticoids for children with COVID-19 routinely (1C) 2) We suggest a low dose and a short duration for severe COVID-19 children only when over inflammatory reaction or in the context of clinical trials. (2D)	100% (Consensus) 93% (Consensus)	11	13
CQ7	We recommend against the use of intravenous immunoglobulin (IVIG) in the treatment of children with severe COVID-19. (1B)	88% (Consensus)	2	2	We recommend against using intravenous immunoglobulin (IVIG) for children with severe COVID-19. (1B)	NA	6	6
CQ8	We propose the following forms of supportive care for children with severe COVID-19: 1) ensuring sufficient number of adequate medical staff in ICUs; 2) systematically monitoring and recording vital signs; 3) using supportive care of the respiratory and cardiovascular symptoms according to clinical needs; 4) providing psychological therapy for children with severe COVID-19	97% (Consensus) 100% (Consensus) 100% (Consensus) 88% (Consensus)	9	9	We propose the following forms of supportive care for children with severe COVID-19: 1) ensuring sufficient number of adequate medical staff in ICUs; 2) systematically monitoring and recording vital signs; 3) using supportive care of the respiratory and cardiovascular symptoms according to clinical needs; 4) providing psychological interventions	NA	5	5
CQ9	We do not recommend interrupting breastfeeding except for mothers with severe COVID-19	72% (Consensus)	15	17	We recommend against mothers interrupting breastfeeding. (2C)	NA	7	7
CQ10	1)We recommend parents to arrange national and international travel with caution during the SARS-CoV-2 epidemic, and follow the epidemiological situation in their travel destination(1D); 2)We recommend parents to obtain information from print media, authorities and official agencies rather than social media(1D) 3)We recommend parents to provide their children with suitable health education to improve the awareness on infectious diseases and teach their children not to discriminate people from areas affected by the epidemic 1D)	94% (Consensus)	8	14	We recommend parents to obtain information regularly from academic and official resources rather than social media. (1D)	NA	2	2

*CQ* Clinical Question, *NA* Not Applicable

^a^Although Recommendation 6 consensus was reached in the first round, a second consensus was conducted in the second round as most experts suggested that it be split into two articles for presentation; The downward slash marks the modified content

### Draft of RAG and external review

According to the Reporting Items for Practice Guidelines in Healthcare (RIGHT) reporting checklist, the RAG development process, clinical questions, recommendations, evidence summary and research gaps, two CMs (Zhou Q, Li W) drafted the first version of the RAG [45, 61]. Four other CMs (Liu E, Luo Z, Smyth RL, Chen Y) made secondary revisions to the content and expression. The revised version of the RAG was reviewed by four invited experts. We selected the external reviewers to ensure a balanced representation of different specialties (one external reviewer was specialized in health policy and guideline methodology, two in pediatric respiratory medicine, and one in pulmonary and critical care medicine), 2geographical regions (one reviewer from the WHO, UK, Chinese mainland and Hong Kong, China each) and genders (one female, three male). All external experts were requested to complete a conflicts of interest form, to identify any factual errors and to comment on the clarity of the language and contextual issues. Finally, the core members discussed and revised the guidelines based on the external reviewers’ comments.

In summary, the development of the RAG by a group of 67 researchers from 11 countries took 50 days from the official commencement of the work (January 28, 2020) to submission (March 17, 2020). The process took another 50 days from submission to acceptance, resulting in a total duration of 100 days (Table 5). In addition, to ensure real-time communication, the development of the RAG was intensively discussed in online meetings almost every week from February 1st to March 7th. A total of 21 meetings between the CM and the RRG were held throughout the RAG development process with a total duration of 48 h (average 2.3 h per meeting) and an average of 16.5 participants attending (Table 6).

**Table 5:** Actual versus planned completion time of the development of RAG

Task	Planned date of completion (day)	Actual date of completion (day)	Excess time spent (days)
Start to work	Jan 28-Jan 28 (One day)	Jan 28-Jan 28 (One day)	0
Write protocol	Jan 29-Jan 29 (One day)	Jan 29-Feb 3 (Six days)	5
Invite panelists	Jan 30-Feb 2 (Four days)	Jan 29-Feb 3 (Six days)	2
Declare conflicts of interests	Jan 30-Feb 2 (Four days)	Jan 30-Feb 5 (Four days)	0
Register guideline	Feb 1-Feb 1 (One day)	Feb 1-Feb 1 (One day)	0
Propose clinical questions	Feb 2-Feb 2 (One day)	Jan 29-Feb 2 (Five days)	4
Select clinical questions	Feb 3-Feb 4 (Two days)	Feb 3-Feb 6 (Four days)	2
Identify PICO clinical questions	Feb 5-Feb 5 (One day)	Feb 7-Feb 15 (Nine days)	8
Retrieve existing systematic reviews	Feb 6-Feb 6 (One day)	Feb 16-Feb 16 (One day)	0
Conduct rapid review^a^	Feb 7-Feb 11 (Five days)	Feb 11-Feb 15(Five days)	0
GRADE evidence	Feb 12-Feb 12 (One day)	Feb 17-Feb 17 (One day)	0
Draft recommendations	Feb 13-Feb 13 (One day)	Feb 18-Feb 23 (Six days)	5
Conduct the 1st round of Delphi survey	Feb 14-Feb 16 (Three days)	Feb 24-Feb 27 (Four days)	1
Conduct the 2ed round of Delphi survey	Feb 17-Feb 19 (Three days)	Feb 28-Mar 1 (Three days)	0
Reach recommendations	Feb 20-Feb 21 (Two days)	Mar 2-Mar 3 (Two days)	0
Draft full guideline	Feb 22-Feb 22 (One day)	Mar 4-Mar 6 (Three days)	2
Send to external reviewers	Feb 23-Feb 24 (Two days)	Mar 6-Mar 15 (Ten days)	8
Revise the guideline	Feb 25-Feb 25 (One day)	Mar 11-Mar 16 (Six days)	5
Submit to medical journal	Feb 26-Feb 26 (One day)	Mar 17 (One day)	0
Accepted by journal	NA	6-May	NA

*NA* Not Applicable

^a^Production of a rapid review in two phases: phases I (Feb 11-Feb 1), just completed the evidence summary sheet; phases II (Feb 16-Apr 14), drafted rapid reviews full-text and submit to journal

**Table 6:** Meetings between Core Members and Rapid Review Group during the RAG development

Number of meetings	Meeting Date	Duration (h)	Participants (number of attendances)	Main Contents of the Meeting	The form of the meeting
1st	Feb-1 (14:30–17:15)	2.75	CM (4), MRRG (22)	1) Selection of the members of expert group 2) Discussion and optimization of initial clinical questions 3) Pre-preparation for the first round of clinical questions (preparation of materials, identification of leaders)	Teleconference – QQ
2nd	Feb-6 (10:30–11:30)	1.0	CM (4), MRRG (20)	1) Feedback from the first round of clinical questions and expert opinion are discussed 2) Preparation of research materials for the second round of clinical questions	Teleconference – QQ
3rd	Feb-7 (16:30–17:30)	1.0	CM (4), MRRG (8)	1) Discussion of late arrangements 2) Identification of final key clinical questions	Teleconference – QQ
4th	Feb-11 (8:30–9:00)	0.5	CM (2), MRRG (24)	1) Discussion of the process of conducting rapid review 2) Identification of search strategies for each clinical question	Teleconference – QQ
5th	Feb-15 (15:00–18:00)	3.0	CM (4), MRRG (22)	1) Discussion of preliminary evidence search results for each clinical question 2) Discussion of preliminary evidence summary 3) Adjustments to the search strategy for several clinical questions (supplementing other indirect evidence)	Teleconference – QQ
6th	Feb-17 (19:00–22:30)	3.5	CM (5), MRRG (24)	1) Discussion of the results of the updated evidence summary 2) Discussion of the results of the adjusted evidence search for clinical questions 3) Drafted recommendations based on evidence summary	Teleconference – QQ
7th	Feb-18 (20:10–22:55)	2.75	CM (5)	1) Modified the evidence summary expression and elaboration for each clinical question 2) Revision of the content of the recommendation	Teleconference – WeChat
8th	Feb-19 (17:00–18:00)	1.0	CM (6), MRRG (10)	1) Modified the evidence summary expression and elaboration for each clinical question 2) Revision of the content of the recommendation	Teleconference – QQ
9th	Feb-20 (14:00–18:45)	4.75	CM (5), MRRG (24)	1) Modified the evidence summary expression and elaboration for each clinical question 2) Revision of the content of the recommendation 3) Preparation of questionnaire materials for the first round of Delphi survey	Teleconference – QQ
10th	Feb-21 (11:00–11:30)	0.5	CM (5)	1) Revision of the wording of the recommendation and evidence summary 2) Revision of the first round of Delphi questionnaire materials	Teleconference – WeChat
11th	Feb-26 (19:00–22:45)	3.75	CM (5), MRRG (23)	1) Discussed the comments from the first round of Delphi survey one by one and revised the content of the recommendations 2) Responded to expert comments and produced feedback report 3) Preparation of questionnaire materials for the second round of Delphi survey	Teleconference – QQ
12th	Feb-29 (9:00–12:00)	3.0	CM (5), MRRG (23)	1) Improved the content of the evidence summary and rationale section 2) Standardizing the format and wording of written content	Teleconference – QQ
13th	Feb-29 (14:00–16:00)	2.0	CM (5), MRRG (23)	1) Improved the content of the evidence summary and rationale section 2) Standardizing the format and wording of written content	Teleconference – QQ
14th	Mar-2 (18:00–19:00)	1.0	CM (6)	1) Discussed the comments from the second round of Delphi one by one and revised the content of the recommendations 2) Responded to expert comments and produced feedback report 3) Discussion of late arrangements (drafting the full guideline)	Teleconference – QQ
15th	Mar-2 (20:00–21:30)	1.5	CM (5)	1) Revision of the content of the recommendations for each clinical question	Teleconference –WeChat
16th	Mar-3 (21:00–22:15)	1.25	CM (5)	1) Revision of the content of the recommendations for each clinical question	Teleconference –WeChat
17th	Mar-4 (18:00–20:15)	2.25	CM (5)	1) Revision of the guidelines based on expert comments 2) Drafting of the full text of the RAG	Teleconference – QQ
18th	Mar-5 (19:00–20:00)	3.0	CM (5), MRRG (10)	1) Revision of the guidelines based on expert comments	Teleconference – QQ
19th	Mar-6 (17:00–20:00)	5.0	CM (6)	1) Identification of journals for submission 2) Identification of external reviewers 3) Revision of the guidelines based on expert comments	Teleconference – QQ
20th	Mar-7 (13:20–15:20)	2.0	CM (3), MRRG (10)	1) Discussed 11 rapid reviews (full text)	Teleconference – QQ
21st	Mar-7 (19:15–21:45)	2.5	CM (5)	1) Revision of the guidelines based on external reviewers’ comments	Teleconference – WeChat
**Total**	NA	48	347	NA	NA
**Average**	NA	2.29 ± 1.30	16.5 ± 10.0	NA	NA

*CM* Core members. The core members include the Chair (Liu E), the Co-Chair (Smyth RL), the Chief Methodologist (Chen Y), the Expert Representative of the Guideline Development Group (Luo Z) and the Leaders of Rapid Review Group (Li W, Zhou Q, Ren L), *MRRG* Members of Rapid Review Group, ***QQ*** QQ is an instant messaging software service and web portal developed by the Chinese tech giant Tencent. QQ offers services that provide online social games, music, shopping, microblogging, movies, and group and voice chat software. *WeChat* WeChat is a Chinese multi-purpose messaging, social media and mobile payment app developed by Tencent, *NA* Not Applicable

### Dissemination and implementation

We have translated this guideline into the following 20 languages: English, Chinese, Japanese, Russian, German, French, Italian, Vietnamese, Thai, Spanish, Arabic, Portuguese, Polish, Czech, Romanian, Burmese, Hungarian, Hebrew, Hindi, Turkish and Malay. The guideline is indexed in the International Practice Guidelines Registry Platform (IPGRP, http://www.guidelines-registry.org/news/141), the Guidelines International Network (GIN, https://g-i-n.net/get-involved/resources/), and the Emergency Care Research Institute (ECRI, https://guidelines.ecri.org/profile/1868) databases. In addition, we contacted the editor-in-chief in advance and discussed publication strategies to ensure a rapid publication (e.g., taking the fast track option, and fast recruitment of peer-reviewers). Considering the differences in health policies and systems, resources, feasibility and equity across the countries, through above strategies, we will assist countries and regions to adopt or adapt the guidelines into their local context.

### Updating plan of the RAG

There is still a lack of effective manuals on how to update the RAG to the standard guideline. Therefore, our working group has proposed the following update plan: 1) According to the WHO guideline handbook [1], when a public health event lasts longer than six months, it should be considered to develop a standard version of the guidelines, and we therefore will update the RAG; 2) The evidence evaluation group will continuously monitor the new evidence from the field of COVID-19 (especially related to children with COVID-19), summarize the new evidence and submit the summaries to the CMs; 3) The CMs will evaluate whether potential new clinical questions should be added in the updated version and the whether the original recommendations are out of date according to the principles proposed by Shekelle et al. [62]; 4) We will invite new clinical experts and methodologists to participate in the updating of the guideline if necessary; 5) We will monitor the updating of existing RAG on COVID-19 and use their experience to guide the updating of our guideline; and 6) The updated guideline will be reported in accordance with the CheckUp checklist [63].

### Challenges in the development process of the RAG

#### Difficulties in collecting and prioritizing clinical questions

As shown in Table 5, it took 50 days to complete the entire development process of the RAG from launch of the guideline to submission to a medical journal. This was 20 days longer than the planned schedule (one month). Two weeks of this time was spent on the identification of clinical questions (proposing clinical questions, selecting the initial clinical questions, prioritizing questions, and formatting them in PICO format).

Because COVID-19 is an emerging infectious disease that was poorly understood in children and even adults when this work was started, it was extremely challenging to select the top ten clinical questions at the beginning of the development of the RAG. First, there was a severe paucity of literature in children on COVID-19 at that time. Second, the limited number of pediatric cases were all contacts of infected adults, and the transmission routes and dynamics among the children themselves or through other sources (e.g. contaminated surfaces) were unknown. Third, the RAG development groups had no frontline clinicians with relevant clinical experience of the natural history of the disease. Fourth, as an international RAG, the clinical questions needed to be applicable to a wide and heterogeneous target population around the world.

The health systems and policies of each country need to be taken into consideration in the process of identifying clinical issues, and the importance of clinical issues vary from country to country. For example, during the survey of clinical questions, the experts proposed the question "who should be tested for nucleic acid at the beginning of an outbreak?". Health policies in some countries have been very clear on this issue (China's policy is to provide free nucleic acid testing to all persons who show clinical signs and have had close contact with a confirmed case [64]). But in most countries, only a limited number of people could be tested in the early stages of the pandemic because of the lack of nucleic acid testing kits.

### Limited of direct evidence

The RAG was supported by 13 rapid reviews that incorporated evidence from studies of COVID-19, SARS and MERS. Table 3 shows only two of the ten recommendations were fully supported by direct evidence for COVID-19, three recommendations were supported by indirect evidence only, and the proportion of COVID-19 studies among the body of evidence in the remaining five recommendations ranged between 10 and 83%. Moreover, only one of the recommendations was fully supported by direct evidence from children with COVID-19, and for seven recommendations there was almost no direct evidence from children with COVID-19.

### Non-peer-reviewed evidence

The RAG aims to provide instructional recommendations in the short term, and therefore studies available as preprints were used to support the evidence. Table 3 shows that six of the ten recommendations used COVID-19 preprints as evidence support, and up to 50% of the studies with direct evidence on COVID-19 were preprints. The RAG recommendations may have been influenced by the variable quality of the underpinning evidence, which may challenge the reliability of the guideline.

### Rapid evolution of evidence

COVID-19 is a global pandemic and the research evidence is growing rapidly. Some of the original studies included in the rapid reviews were retracted for various reasons, resulting in the data being updated again during the production of the rapid review. For example, two studies included in the rapid review for the clinical question #4 (“Should Antiviral drugs such as ribavirin, interferon, remdesivir (GS-5734), lopinavir /ritonavir or oseltamivir be used to treat children with COVID-19?") were withdrawn, a controlled study on favipiravir was temporarily removed from *Engineering* on April 1 [65], and another non-randomized controlled trial of hydroxychloroquine was withdrawn from the *International Journal of Antimicrobial Agents* on April 3rd due to not meeting the industry consensus criteria [66]. Because of the withdrawal of these studies, the rapid reviews had to be updated and revised.

### Strengths in the development process of the RAG

#### Prospective registration and publication of the guideline protocol

The registration and publication of the protocol determines in advance the steps and methodology to be followed in developing the RAG according to the WHO handbook. Thorough development and documentation of the methodology, key issues and outcomes reduces the bias in the formulation of recommendations and minimizes the risk of inducing randomness into decision-making. In addition to the above, the registration of the guideline helps to increase the transparency of the development process, avoid duplication, enhance the credibility of the guidelines, and facilitate their dissemination and implementation.

### Considering parental preferences and values

The collection and integration of parents’ preferences and values to form recommendations will help families fully understand the pros and cons of various treatment options, so that they have a stronger sense of acceptance and willingness to participate in clinical decision-making. Consideration of families’ preferences and values can thus improve patient compliance, maximize patient benefits, and improve their clinical outcomes [67]. Therefore, the RAG conducted a recommendation survey process that incorporated two guardians of children scoring our recommendations and providing relevant feedback.

### Collection and prioritization of clinical questions

Clinical questions are the starting point for guideline development that determine the scope of evidence to be retrieved and evaluated at a later stage. Clinical questions also determine the content of the final recommendations [68]. Due to the urgency of the COVID-19 public health situation, solving all clinical problems at once was not possible, so we conducted a Delphi survey to rank clinical problems by a representative group of experts, and finalized 10 key clinical questions for clinicians fighting the epidemic in the frontline.

### Evidence-based recommendations based on rapid review

The development of guidelines must consider the best currently available research evidence to ensure that the recommendations are comprehensive and objective. Rapid reviews can synthesize the available research evidence on the same topic in a short duration of time, and thus reduce the risk of bias and ultimately improve the reliability and accuracy of decision making. Our guideline was accompanied by 13 rapid reviews to support the development of recommendations.

### Advantages of following the manual and protocol

It is well known that strict adherence to guideline development manuals and the protocol can enhance the transparency and clarity and ensure the quality and credibility of the guideline [69]. In this particular case, we did not use other standard handbooks due to time constraints. We registered a protocol, and followed the protocol and the WHO RAG manual in every step of the development process. The manual is specifically intended for rapid advice guidelines, and contains several aspects that help to carry out the development in a rapid and efficient manner. The recommendations of the WHO RAG handbook differs from the standard guideline development in the following ways:1) The method of rapid systematic review production is used instead of the traditional development process of systematic reviews in evidence synthesis, which helps to save a lot of time in document screening, evaluation and synthesis; 2) Online meetings are used instead of face-to-face meetings, which saves time, administrative workload and resources of both the organizers and the attending experts; and 3) the WHO RAG handbook requires all members of the expert group to prioritize the preparation of the guidelines, postpone other non-urgent matters, and provide efficient feedback around the rapid development of the guidelines, which guarantees a rapid turnaround time of thefeedback and response.

## Conclusion

In order to respond to public health emergencies, the development of a RAG requires a clear and transparent formulation process, and usually uses a large amount of indirect and non-peer-reviewed evidence to support the formation of recommendations. Strictly following the WHO RAG handbook does not only enhance the transparency and clarity of the guideline, but also speeds up the guideline development process, thereby saving time and resources.

## Supplementary Information

**Additional file 1.**

**Additional file 2.**

**Additional file 3.**

## Data Availability

All data generated or analyzed during this study are included in this published article and its additional file.
