# Supplementary material for: Methodology and experiences of rapid advice guideline development for children with COVID-19: responding to the COVID-19 outbreak quickly and efficiently
Source: BMC Med Res Methodol. 2022 Apr 3;22:89. doi: 10.1186/s12874-022-01545-5 (PMC8977048; doi:10.1186/s12874-022-01545-5)
Supplement: Supplementary file 1 — Additional file 1. [file 12874_2022_1545_MOESM1_ESM.docx]

**Methodology and** **Experiences of Rapid Advice Guideline Development for Children with COVID-19: Responding to the COVID-19 outbreak quickly and efficiently**

**Qi Zhou^1,2^****^#^, Qinyuan Li^3#^, Janne Estill^4,5^, Qi Wang^6,7^, Zijun Wang^1^, Qianling Shi^8^, Jingyi Zhang^9^, Xiaobo Zhang^10^, Joseph L. Mathew^11^, Rosalind L. Smyth^12^, Detty Nurdiati^13^, Zhou Fu^3^, Hongmei Xu^14^, Xianlan Zheng^15^, Xiaodong Zhao^16^, Quan Lu^17^, Hui Liu^9^, Yangqin Xun^1^, Weiguo Li^3^, Shu Yang^18^, Xixi Feng^19^, Mengshu Wang^20^, Junqiang Lei^20^, Xiaoping Luo^21^, Liqun Wu^22^, Xiaoxia Lu^23^, Myeong Soo Lee^24,25,26^, Shunying Zhao^27^, Edwin Shih-Yen Chan^28,29^, Yuan Qian^30^, Wenwei Tu^31^, Xiaoyan Dong^32^, Guobao Li^33,34^, Ruiqiu Zhao^14^, Zhihui He^35^, Siya Zhao^9^, Xiao Liu^9^, Qiu Li^36^, Kehu Yang^1,37,38^,** **Zhengxiu Luo^3*^, Enmei Liu^3*^, Yaolong Chen^1,38,39,40,4^****^1*^**

1. *Evidence-based Medicine Center, School of Basic Medical Sciences, Lanzhou University, Lanzhou 730000, China;*
2. *Lanzhou University Institute of Health Data Science, Lanzhou, China;*
3. *Department of Respiratory Medicine Children’s Hospital of Chongqing Medical University, National Clinical Research Center for Child Health and Disorders, Ministry of Education Key Laboratory of Child Development and Disorders, Chongqing Key Laboratory of Pediatrics, Chongqing, China;*
4. *Institute of Global Health, University of Geneva, Geneva, Switzerland;*
5. *Institute of Mathematical Statistics and Actuarial Science, University of Bern, Bern, Switzerland;*
6. *Department of Health Research Methods, Evidence and Impact, Faculty of Health Sciences, McMaster University, Hamilton, Canada;*
7. *McMaster Health Forum, McMaster University, Hamilton, Canada;*
8. *The First School of Clinical Medicine, Lanzhou University, Lanzhou, China;*
9. *School of Public Health, Lanzhou University, Lanzhou, China;*
10. *Children’s Hospital of Fudan University, National Children's Medical Center, Shanghai, China;*
11. *Advanced Pediatrics Centre, PGIMER Chandigarh, Chandigarh, India;*
12. *UCL Great Ormond Street Institute of Child Health, London, UK;*
13. *Cochrane Indonesia, Faculty of Medicine, Public Health and Nursing, Universitas Gadjah Mada, Yogyakarta, Indonesia;*
14. *Department of Infection Diseases Children's Hospital of Chongqing Medical University, National Clinical Research Center for Child Health and Disorders, Ministry of Education Key Laboratory of Child Development and Disorders, Chongqing Key Laboratory of Pediatrics, Chongqing, China;*
15. *Department of Nursing Children's Hospital of Chongqing Medical University, National Clinical Research Center for Child Health and Disorders, Ministry of Education Key Laboratory of Child Development and Disorders, Chongqing Key Laboratory of Pediatrics, Chongqing, China;*
16. *Department of Pediatric Research Institute, Ministry of Education Key Laboratory of Child Development and Disorders, National Clinical Research Center for Child Health and Disorders, China International Science and Technology Cooperation Base of Child Development and Critical Disorders, Children's Hospital of Chongqing Medical University, Chongqing, China;*
17. *Shanghai Children’s Hospital affiliated to Shanghai Jiaotong University, Shanghai, China;*
18. *Digital Institute of Medicine, Chengdu University of Traditional Chinese Medicine, Chengdu, China;*
19. *Department of Public Health, Chengdu Medical College, Chengdu, China;*
20. *Department of Radiology, the First Hospital of Lanzhou University, Lanzhou, China;*
21. *Department of Pediatrics, Tongji Hospital, Tongji Medical College, Huazhong University of Science and Technology, Wuhan, China;*
22. *Shenzhen Health Development Research Center, Shenzhen, China;*
23. *Department of Respiratory Medicine, Wuhan Children’s Hospital, Tongji Medical College, Huazhong University of Science and Technology, Wuhan, China;*
24. *Clinical Medicine Division, Korea Institute of Oriental Medicine, Daejeon, S. Korea*
25. *Korean Convergence Medicine, University of Science and Technology, Daejeon, S. Korea*
26. *Tianjin University of Traditional Chinese Medicine, Tianjin, China*
27. *Beijing Children’s Hospital, Beijing, China;*
28. *Centre for Quantitative Medicine, Office of Clinical Sciences, Duke-National University of Singapore Medical School, Singapore;*
29. *Singapore Clinical Research Institute, Singapore;*
30. *Beijing Key Laboratory of Etiology of Viral Diseases in Children, Capital Institute of Pediatrics, Beijing, China;*
31. *Department of Pediatrics & Adolescent Medicine, Li Ka Shing Faculty of Medicine, University of Hong Kong, Hong Kong, China;*
32. *Shanghai Children’s Hospital, Shanghai, China;*
33. *National Clinical Research Center for Infectious Disease, Shenzhen, China;*
34. *Shenzhen Third People’s Hospital, Shenzhen, China;*
35. *Chongqing Ninth People’s Hospital, Chongqing, China;*
36. *Department of Nephrology Children’s Hospital of Chongqing Medical University, National Clinical Research Center for Child Health and Disorders, Ministry of Education Key Laboratory of Child Development and Disorders, Chongqing Key Laboratory of Pediatrics, Chongqing, China;*
37. *Key Laboratory of Evidence Based Medicine & Knowledge Translation of Gansu Province, Lanzhou, China;*
38. *WHO Collaborating Centre for Guideline Implementation and Knowledge Translation, Lanzhou, China;*
39. *GIN Asia, Lanzhou 730000, China;*
40. *Lanzhou GRADE Centre, Lanzhou 730000, China;*
41. *Lanzhou University, an Affiliate of the Cochrane China Network, Lanzhou 730000, China.*

***#These authors contributed equally to this work.***

**Correspondence to: Yaolong Chen. Evidence-based Medicine Center, School of Basic Medical Sciences, Lanzhou University, Lanzhou, China. Email: sinograde@163.com; Enmei Liu. Department of Respiratory Medicine Children’s Hospital of Chongqing Medical University, National Clinical Research Center for Child Health and Disorders, Ministry of Education Key Laboratory of Child Development and Disorders, Chongqing Key Laboratory of Pediatrics, Chongqing, China; Email: emliu186@126.com; Zhengxiu Luo. Department of Respiratory Medicine Children’s Hospital of Chongqing Medical University, National Clinical Research Center for Child Health and Disorders, Ministry of Education Key Laboratory of Child Development and Disorders, Chongqing Key Laboratory of Pediatrics, Chongqing, China; Email: luozhengxiu816@163.com.*

**Additional file 1: Guideline Working Group**

***Chair***

**Professor Enmei Liu, Pediatric Pulmonologist**

National Clinical Research Center for Child Health and Disorders (Chongqing); Ministry of Education Key Laboratory of Child Development and Disorders;

China International Science and Technology Cooperation base of Child development and Critical Disorders;

Children’s Hospital of Chongqing Medical University, Chongqing, China

E-mail: emliu186@126.com

***Co-chair***

**Professor Rosalind L** **Smyth - Co-chair of the Guideline Group, Respiratory**

**Director UCL Great Ormond St Institute of Child Health, London, United Kingdom**

**Honorary Consultant Respiratory Paediatrician, Great Ormond Street Hospital, London**

**E-mail: rosalind.smyth@ucl.ac.uk**

***Chief Methodologist***

**Professor Yaolong** **Chen, Chief methodologist, Methodologist**

**Evidence Based Medicine Centre of Lanzhou University, Gansu, China**

**WHO Collaborating Centre for Guideline Implementation and Knowledge Translation**

**Chair, GIN Asia**

**Director, Chinese GRADE Centre**

**Head, Guideline Working Group of Cochrane China Network**

**E-mail: chenyaolong@vip.163.com**

***Academic and Executive Administrator***

**Professor Qiu Li, Academic and executive administrator, Nephrologist and Immunologist**

National Clinical Research Center for Child Health and Disorders (Chongqing); Ministry of Education Key Laboratory of Child Development and Disorders;

China International Science and Technology Cooperation base of Child development and Critical Disorders;

Children’s Hospital of Chongqing Medical University, Chongqing, China

E-mail: liqiu809@126.com

***Guideline Development Group***

**Professor Zhengxiu Luo, Pediatric Pulmonologist**

National Clinical Research Center for Child Health and Disorders (Chongqing); Ministry of Education Key Laboratory of Child Development and Disorders;

China International Science and Technology Cooperation base of Child development and Critical Disorders;

Children’s Hospital of Chongqing Medical University, Chongqing, China

E-mail: luozhengxiu816@163.com

**Dr. Amir Qaseem, Vice President**

Clinical Policy and Center for Evidence Reviews, American College of Physicians

Guidelines International Network, US

E-mail: aqaseem@acponline.org

**Professor Joseph L. Mathew, Professor (Pediatric Pulmonology)**

Advanced Pediatrics Centre, PGIMER Chandigarh, India

E-mail: joseph.l.mathew@gmail.com

**Professor Quan Lu, Paediatric Pulmonologist**

Shanghai Children’s Hospital affiliated to Shanghai Jiaotong University, Shanghai, China

E-mail: luquan-sh@vip.sina.com

**Dr. Wilson Milton Were, Medical Officer**

Maternal, Newborn, Child and Adolescent Health & Ageing, WHO

E-mail: werew@who.int

**Dr. Mansuk Daniel HAN, Medical Officer**

Maternal, Newborn, Child and Adolescent Health & Ageing, WHO

E-mail: mhan@who.int

**Professor Zhou Fu, Pediatric Pulmonologist**

Children's Hospital of Chongqing Medical University

National Clinical Research Center for Child Health and Disorders (Chongqing); Ministry of Education Key Laboratory of Child Development and Disorders;

China International Science and Technology Cooperation base of Child development and Critical Disorders;

Children’s Hospital of Chongqing Medical University, Chongqing, China

E-mail: fu_zhou79@aliyun.com

**Professor Xiaodong Zhao, Immunologist**

National Clinical Research Center for Child Health and Disorders (Chongqing); Ministry of Education Key Laboratory of Child Development and Disorders;

China International Science and Technology Cooperation base of Child development and Critical Disorders;

Children’s Hospital of Chongqing Medical University, Chongqing, China

E-mail: zhaoxd530@aliyun.com

**Professor Shunying Zhao, Pediatric Pulmonologist**

Beijing Children's Hospital, Beijing, China

E-mail: zhaoshunying2001@163.com

**Dr. Janne Estill, Senior Researcher**

Institute of Global Health, University of Geneva, Switzerland

Institute of Mathematical Statistics and Actuarial Science, University of Bern, Switzerland

E-mail: janne.estill@stat.unibe.ch

**Professor Edwin Shih-Yen Chan, Director and Chief Scientific Officer**

Centre for Quantitative Medicine, Office of Clinical Sciences, Duke-National University of Singapore Medical School

Singapore Clinical Research Institute, Singapore

E-mail: edwin.chan@scri.edu.sg

**Dr. Lei Liu, Pediatric Infectious Disease Specialist**

National Clinical Research Center for Infectious Disease

Shenzhen Third People’s Hospital, Shenzhen, China

E-mail: Liulei3322@aliyun.com

**Professor Yuan Qian, Pediatric Infectious Disease Specialist**

Capital Institute of Pediatrics, China

E-mail: yqianbjc@263.net

**Professor Hongmei Xu, Pediatric Infectious Disease Specialist**

National Clinical Research Center for Child Health and Disorders (Chongqing); Ministry of Education Key Laboratory ofChild Development and Disorders;

China International Science and Technology Cooperation base of Child development and Critical Disorders;

Children’s Hospital of Chongqing Medical University, Chongqing, China

E-mail: xuhongm0095@sina.com

**Dr. Qi Wang, Health Policy Researcher**

Department of Health Research Methods, Evidence and Impact, Faculty of Health Sciences, McMaster University, Canada

McMaster Health Forum, McMaster University, Canada

E-mail: wangq87@mcmaster.ca

**Professor Toshio Fukuoka, Chief Director**

Emergency and Critical Care Center, the Department of General Medicine

Department of Research and Medical Education at Kurashiki Central Hospital

Advisory Committee in Cochrane Japan, Japan

E-mail: tf11308@kchnet.or.jp

**Professor Xiaoping Luo, Director Paediatrician**

Department of Pediatrics, Tongji Hospital, Tongji Medical College, Huazhong University of Science and Technology, Wuhan, China

E-mail: xpluo@tjh.tjmu.edu.cn

**Professor Wong Wing Kin Gary, Pediatric Pulmonologist**

Department of Pediatrics, The Chinese University of Hong Kong, China Hongkong

E-mail: wingkinwong@cuhk.edu.hk

**Professor Junqiang Lei, Radiologist**

The First School of Clinical Medicine, Lanzhou University, Lanzhou, China

E-mail: leijq1990@163.com

**Dr. Detty Nurdiati, Methodologist**

Clinical Epidemiology & Biostatistics Unit (CEBU), Div. of Maternal Fetal Medicine, Dept. of Obstetrics & Gynecology, Fac of Medicine/Dr. Sardjito Hospital Universitas Gadjah Mada, Yogyakarta, Indonesia

E-mail: dnurdiati@yahoo.com

**Professor Wenwei Tu, Pediatric Infectious Disease Specialist**

Department of Paediatrics& Adolescent Medicine, Li Ka Shing Faculty of Medicine, University of Hong Kong, Hong Kong SAR, China

E-mail: mailto:wwtu@hku.hk

**Professor Xiaobo Zhang, Pediatrics General Specialist**

Children's Hospital of Fudan University, Shanghai, China

E-mail: zhangxiaobo0307@163.com

**Professor Xianlan Zheng, Nurse Specialist**

Nursing Department, Children’s Hospital of Chongqing Medical University

E-mail: zhengxianlan@cqmu.edu.cn

**Professor Hyeong SikAhn, Director, Evidence-based Medicine Researcher**

Department of Preventive Medicine, Korea University, Korea

Korea Cochrane Centre

Evidence Based Medicine, Korea

Korea University School of Medicine

E-mail: ahnhann@gmail.com

**Dr. Mengshu Wang, Radiologist**

The First School of Clinical Medicine, Lanzhou University, Lanzhou, China

E-mail: 251291442@qq.com

**Dr. Xiaoyan Dong, Director Paediatrician**

Shanghai Children's Hospital, Shanghai, China

E-mail: dong_x_y0305@126.com

**Dr. Liqun Wu, Minister, Health economics expert**

Shenzhen health development research center, Shenzhen, China

E-mail: 57128241@qq.com

**Professor Myeong Soo Lee, Principal Researcher**

Korea Institute of Oriental Medicine, Daejeon, S. Korea

University of Science and Technology, Daejeon, S. Korea

London Southbank University, London, UK

Tianjin University of Traditional Chinese Medicine, Tianjin, China

E-mail: drmslee@gmail.com

**Dr. Guobao Li, Infectious Disease Specialist**

National Clinical Research Center for Infectious Disease

Shenzhen Third People’s Hospital, Shenzhen, China

E-mail: feisanke-01@szsy.sustech.edu.cn

**Professor Shu Yang, Statistician**

Chengdu University of TCM, Chengdu, China

E-mail: sishiyu1978@qq.com

**Professor Xixi Feng, Epidemiologist**

Chengdu Medical College, Chengdu, China

E-mail: 583840943@qq.com

**Dr. Ruiqiu Zhao, Pediatric Infectious Disease Specialist**

National Clinical Research Center for Child Health and Disorders (Chongqing); Ministry of Education Key Laboratory of Child Development and Disorders; China International Science and Technology Cooperation base of Child development and Critical Disorders; Children’s Hospital of Chongqing Medical University, Chongqing, China

E-mail: zrq0907@yeah.net

**Professor Xiaoxia Lu, Pediatric Pulmonologist**

Department of Respiratory Medicine, Wuhan Children’s Hospital, Tongji Medical College, Huazhong University of Science and Technology, Wuhan, Hubei, China

E-mail: Lusi74@163.com

**Dr. Zhihui He, Primary Care Paediatrician Specialist**

Chongqing ninth people's hospital, Chongqing, China

E-mail: hezhihui726@sina.com

**Dr. Shihui Liu, Senior Lawyer**

Beijing Jishuitan Hospital, Beijing, China

E-mail: 13811790161@163.com

***Leaders of Rapid Review Group***

**Dr. Weiguo Li, Primary Care Paediatrician Specialist**

Children's Hospital of Chongqing Medical University

National Clinical Research Center for Child health and disorders, Chongqing, China

E-mail: lwgyanda@gmail.com

**Dr. Qi Zhou, Master Student**

The First School of Clinical Medicine, Lanzhou University, Lanzhou, China

E-mail: zhouq18@lzu.edu.cn

**Dr. Luo** **Ren, Virology Researcher**

Children's Hospital of Chongqing Medical University

National Clinical Research Center for Child health and disorders, Chongqing, China

E-mail: luo.ren@ucl.ac.uk

***Patient Representatives***

**Mr.** **Xingxing Wu, Child patient guardian**

A guardian of a child patient of the Third People's Hospital of Shenzhen

**Mr. Xingli Zhou, Child patient guardian**

A guardian of a child patient of the Children's Hospital of Chongqing Medical University

**Note:** Core members (CM). The core members include the Chair (Liu E), the Co-Chair (Smyth RL), the Chief Methodologist (Chen Y), the Expert Representative of the Guideline Development Group (Luo Z) and the Leaders of Rapid Review Group (Li W, Zhou Q, Ren L)
