# Supplementary material for: Methodology and experiences of rapid advice guideline development for children with COVID-19: responding to the COVID-19 outbreak quickly and efficiently
Source: BMC Med Res Methodol. 2022 Apr 3;22:89. doi: 10.1186/s12874-022-01545-5 (PMC8977048; doi:10.1186/s12874-022-01545-5)
Supplement: Supplementary file 2 — Additional file 2. [file 12874_2022_1545_MOESM2_ESM.docx]

**Additional file 2：List of 13 rapid reviews**

1. Nussbaumer-Streit B, Mayr V, Dobrescu AI, et al. Quarantine alone or in combination with other public health measures to control COVID-19: a rapid review. Cochrane Database Syst Rev. 2020;4:CD013574. doi:10.1002/14651858.CD013574
2. Wang Z, Zhou Q, Wang C, et al. Clinical characteristics of children with COVID-19: a rapid review and meta-analysis. Ann Transl Med. 2020;8:620. doi:10.21037/atm-20-3302
3. Gao Y, Liu R, Zhou Q, et al. Application of telemedicine during the coronavirus disease epidemics: a rapid review and meta-analysis. Ann Transl Med. 2020;8:626. doi:10.21037/atm-20-3315
4. Zhou Q, Gao Y, Wang X, et al. Nosocomial infections among patients with COVID-19, SARS and MERS: a rapid review and meta-analysis. Ann Transl Med. 2020;8:629. doi:10.21037/atm-20-3324
5. Lv M, Wang M, Yang N, et al. Chest computed tomography for the diagnosis of patients with coronavirus disease 2019 (COVID-19): a rapid review and meta-analysis. Ann Transl Med. 2020;8:622. doi:10.21037/atm-20-3311
6. Shi Q, Zhou Q, Wang X, et al. Potential effectiveness and safety of antiviral agents in children with coronavirus disease 2019: a rapid review and meta-analysis. Ann Transl Med. 2020;8:624. doi:10.21037/atm-20-3301
7. Wang J, Tang Y, Ma Y, et al. Efficacy and safety of antibiotic agents in children with COVID-19: a rapid review. Ann Transl Med. 2020;8:619. doi:10.21037/atm-20-3300
8. Lu S, Zhou Q, Hang L, et al. Effectiveness and safety of glucocorticoids to treat COVID-19: a rapid review and meta-analysis. Ann Transl Med. 2020;8:627. doi:10.21037/atm-20-3307
9. Zhang J, Yang Y, Yang N, et al. Effectiveness of intravenous immunoglobulin for children with severe COVID-19: a rapid review. Ann Transl Med. 2020;8:625. doi:10.21037/atm-20-3305
10. Luo X, Lv M, Wang X, et al. Supportive care for patient with respiratory diseases: an umbrella review. Ann Transl Med. 2020;8:621. doi:10.21037/atm-20-3298
11. Yang N, Che S, Zhang J, et al. Breastfeeding of infants born to mothers with COVID-19: a rapid review. Ann Transl Med. 2020;8:618. doi:10.21037/atm-20-3299
12. Li W, Liao J, Li Q, et al. Public health education for parents during the outbreak of COVID-19: a rapid review. Ann Transl Med. 2020;8:628. doi:10.21037/atm-20-3312
13. Jefferson T, Del Mar CB, Dooley L, et al. Physical interventions to interrupt or reduce the spread of respiratory viruses. Cochrane Database Syst Rev. 2011;2011:CD006207. doi:10.1002/14651858.CD006207.pub4
