# Supplementary material for: Methodology and experiences of rapid advice guideline development for children with COVID-19: responding to the COVID-19 outbreak quickly and efficiently
Source: BMC Med Res Methodol. 2022 Apr 3;22:89. doi: 10.1186/s12874-022-01545-5 (PMC8977048; doi:10.1186/s12874-022-01545-5)
Supplement: Supplementary file 3 — Additional file 3. [file 12874_2022_1545_MOESM3_ESM.docx]

**Additional file 3: Panelists Comments Feedback Reports**

**Panelists Comments Feedback Report on First Round Recommendations Survey**

**Notes:** As far to February 27th, 2020, the first-round survey of recommendations on the guidelines had collected questionnaire results from 33 panelists. The survey results and their feedback have been added in the following table.

1. The comments of panelists have been replied. For details, please refer to "Panelists comments and Reply" in each clinical question.

2. The revised recommendation have been marked with red.

3. We present the level of agreement of each recommendation opinion (level of agreement = the number of panelists agreed / the total number of panelists participating in the survey * 100%).

4. Q: Question; E: Panelists Comments; R: Reply.

| **Clinical question 1: What are the main clinical features of children with COVID-19?** | |
| --- | --- |
| **Preliminary recommendation:** | **Level of Agreement** |
| - **The main symptoms of children with COVID-19 are fever and cough. The symptoms of COVID-19 are usually less severe in children than adults. Leukocyte and lymphocyte counts are usually normal. Although there is no significant difference between adults and children in chest imaging characteristics, the extend of the abnormalities are usually less in children. (2C)** | **Agree: 31 (94%)**  **Disagree: 1 (3%)**  **Unsure: 1 (3%)** |
| **Panelists Comments and Reply**  **This clinical question has received 11 comments from 10 panelists, our reply to the feedback is as follows：**  **Q1:** On the problem of “inconsistent format of references”.   - E1: Inconsistent format of references.   **R1:** We have revised the references according to the periodical format according to the experts' opinions after discussion.  **Q2:** On the problem of “the exact expression of ‘children with COVID-19’ and ‘children with SARS-CoV-2 infection’”.   - E1: Should we change “children with COVID-19 are more common to have only mild symptoms" into "Children with SARS-CoV-2 infection are more common to have mild COVID-19"？ Because they are not just mild symptoms, but less severe diseases caused by viral infection.   **R2:** We have not revised the disease name and COVID-19 was still adopted after discussion.  **Q3:** On the problem of“the explanation is based on the conclusion of limited child cases”.   - E1: We should clarify that this is based on experiences in a limited number of children.   **R3:** We have revised our rationale and changed " Based on published case series " into " Based on published case series with a limited number of children".  **Q4:** On the problem of“whether to explain the unusual clinical symptoms”.   - E1: Some uncommon symptoms such as diarrhea or vomiting should be mentioned.   **R4:** We have revised our evidence by adding a case report that started with gastrointestinal symptoms.  **Q5:** On the problem of“whether to emphasize the large proportion of asymptomatic infection in children's symptoms”.   - E1: There is large proportion of asymptomatic infection in children. Should we emphasize it here? - E2: It could be also mentioned that a large part of children had no symptoms, and the large majority had only one symptom (unlike adults).   **R5:** We have revised our rationale and evidence by further emphasizing the proportion of asymptomatic children (about 23%).  **Q6:** On the problem of“abnormal image range of children ”.   - E1：Although chest imagining between infected children and adults are similar, the extend of the abnormalities are usually less in children. - E2：There is insufficient evidence as the last sentence saying "there is no obvious difference between the image performance of adults and children". Only 15 children in Shenzhen showed atypical chest image performance, mainly ground-glass density image.   **R6:** We have revised our recommendation and added related evidence and explanation in the part of evidence. Refer to the Delphi survey for specific recommendations.  **Q7:** On the problem of“the logical order of the presentation of children's symptoms ”.   - E1：The table is good, however, a little confused. 1) mild symptoms, severe symptoms, and no symptom, more than one, I think they are the same category; 2) fever, cough, they are another category, they belong to the symptoms. Should we separate the table? 3) should we rank the symptoms as a certain order (such as the frequency in children?)   **R7:** We have revised the order of the tables.  **Q8:** On the problem of“present children's imaging features in the recommendations”.   - E1：The chest imaging findings should be elaborated by adding the following: Chest imaging findings of unilateral or bilateral ground-glass shadow do not significantly differ between adults and children.   **R8:** We have revised our rationale and evidence by adding and explaining the imaging features of children.  **Q9:** On the problem of“whether the evidence retrieval of severe cases in children is sufficient ”.   - E1：There is only one child with severe illness, and it is unknown whether the evidence retrieval is sufficient.   **R9:** We confirmed that there is only one serious case reported currently. | |

| **Clinical question 2: How should children suspected with SARS-CoV-2 infection be managed?** | |
| --- | --- |
| **Preliminary recommendation** | **Level of Agreement** |
| - **We suggest that children who have been in close contact with COVID-19 patients are initially evaluated by their guardians or family doctors. If no obvious symptoms occur, we recommend staying at home for observation for a duration of at least 14 days; if there are obvious symptoms, we suggest further evaluation in the hospital. (2C)** | **Agree: 31（94%）**  **Disagree: 0（0%）**  **Unsure: 2（6%）** |
| **Panelists Comments and Reply**  **This clinical question has received 11 comments from 9 panelists, our reply to the feedback is as follows:**  **Q1:** On the problem of "fever clinic and recommendation intensity".   - E1：we suggest further evaluation in the hospital with fever clinics；Replace "2C" with "1C"。 - E2：if it is weak recommendation (2c), should we say "we suggest" rather than "we recommend"? The same question is also for other recommendations, such as Recommendation 3.   **R1:** We have revised our recommendation based on existing evidences. Our guideline is a global guideline, considering that not all countries and regions have the conditions for fever clinic, it has not been modified. As for the determination of the recommended intensity, we will make unified adjustment based on the evidence and factors affecting the recommended intensity in the later stage. We have revised the recommendations based on the replies. Refer to the Delphi survey for specific recommendations.  **Q2:** On the problem of "the proportion of asymptomatic children is inconsistent".   - E1: question-1 says that 28% of the children have no symptoms, and the literature here says that 34% of the children have no symptoms - inconsistent!   **R2:** We have revised our recommendation based on existing evidences. At present, the data of the proportion of asymptomatic children quotes from the unpublished data of the research group. And we will continue to quote accurate data and keep consistent with clinical question 1.  **Q3:** On the problem of "change ‘withCOVID-19’ into ‘with COVID-19’".   - E1：“withCOVID-19 ”change into "with COVID-19"。   **R3:** We have revised and changed "withCOVID-19" into "with COVID-19".  **Q4:** On the problem of home observation duration   - E1；Joseph L. Mathew：we recommend staying at home for observation for a duration of at least xx days. If symptoms appear, then ,……………………… .   **R4:** We have revised our recommendation and changed "we recommended staying at home for observation" into "we recommended staying at home for observation for a duration of at least 14 days". Refer to the Delphi survey for specific recommendations.  **Q5:** On the problem of " symptom description ".   - E1：If the children who have been in close contact with COVID-19 patients have any symptoms, including respiratory tract and disgestive tract symptoms, further evaluation in the hospital should be considered. - E2：the highlighted part "such as fever and cough", the word "and", could it be replaced with "and/or"? - E3：should we explain the highlighted sentence in the "rational" part? how to judge to go home or wait in hospitals depending on what kinds of conditions?   **R5:** We have revised our recommendation based on existing evidences. The symptom description part has been directly summarized as "obvious symptoms", and the gastrointestinal symptoms and respiratory symptoms have been described in the rationale part.  **Q6:** On the problem of " nucleic acid detection".   - E1: I think that the children who have been in close contact with COVID-19 patients should be at home for Isolation observation if noobvious symptoms are found. They should do a SARS-CoV-2 nucleic acid test as soon as possible, because there are much more asymptomatic adult and children with SARS-CoV-2 infected by far.   **R6:** We haven't revised our recommendation based on the following reasons. Firstly, according to the medical conditions of different countries, active nucleic acid testing for children with contact history will increase the medical burden. Secondly, it has been reported that the new coronavirus nucleic acid testing could get false negative result.  **Q7:** On the problem of “Home isolation and protection”.   - E1: is it recommended that during home isolation, the child's caregiver should pay attention to personal protection and avoid contact with others (self-isolation).   **R7:** We haven't revised our recommendation based on existing evidences. According to the current evidence, infection in children is mostly caused by family clustering infection, and is mostly infected by their family members. Asymptomatic children are less likely to infect adults, and children wouldn’t get enough care if they were isolated.  **Q8:** On the problem of " relevance of cited evidence and guidelines".   - E1: Unsure whether the evidence cited is relevant to the clinical question.   **R8:** We haven't revised our recommendation because there is no direct evidence for children at present, we quoted the relevant novel coronavirus research of adults and children with SARS or MERS as evidence, which have indirect association to some degree and could provide certain values for our decision-making. | |

| **Clinical question 3: Can chest imaging test help diagnose children with COVID-19?** | |
| --- | --- |
| **Preliminary recommendation:** | **Level of Agreement** |
| - **We suggest not using imaging test as routine examination for children with COVID-19. (2C)** | **Agree: 18 (55%)**  **Disagree: 10 (30%)**  **Unsure: 5 (15%)** |
| **Panelists Comments and Reply**  **This clinical question has received 12 comments from 32 Panelists, our reply to the feedback is as follows**  **Q1:** On the problem of “The evidence for using X-ray as auxiliary examination isn’t strong enough”.   - E1: At present, there is insufficient evidence for using radiological examination to diagnose children with COVID-19, and it is recommended that clinicians make decisions after weighing the risks and benefits according to clinical conditions. - E2: The recommendation sounds still very positive towards X-ray. The evidence shows no clear benefit for X-ray imaging in children, so the only evidence is indirect from adults. The recommendation could be formulated more carefully, e.g. that X-ray should be used only if the results of other tests were indeterminate; and CT scan never. - E3: The evidence presented does not support the recommendation that chest x-ray rather than CT should be used. - E4: Second，I did not find sufficient evidence to support chest X-rays in children with COVID-19, On the one hand, children are mostly mild, can X-rays detect symptoms? On the other hand, milder symptoms mean that medical observation should be given. For these children, whether they are diagnosed or not will not affect treatment   **R1:** We have revised our recommendation based on existing evidences. Refer to the Delphi survey for specific recommendations  **Q2:** On the problem of “ Recommend using CT in children with COVID-19”.   - E1:The clinical feature of pneumonia among children with COVID-19 is less typical compared to adults. Use of X-ray has limitations since no foci can be observed at early stage. The pneumonia would be very severe if the X-ray is positive. COVID-10 is highly contagious, CT is sensitive to viral pneumonia, thus children should be quarantined and wait for nucleic acid test results once their CT show signs of viral pneumonia. There are cases where nucleic acid test result turns positive at the sixth test, thus CT as accessory examination cannot be exempted. Children could consider low dose CT. For those who can't be ruled out by PCR (PCR negative) for the first time, conduct additional examination with low dose CT. If CT result shows signs of viral pneumonia, implement quarantine and conduct PCR for a second time. - E2: Suggesting CT as auxiliary examination. - E3: X-ray examination is not better than CT if the child was suspected with COVID-19 infected. Because CT is higher sensitivity than X ray. Children who were suspected should do CT examination as soon as possible , in order to confirm pneumonia early and treat in hospital in time - E4: Chest CT better than plain radiography. Early lung changes are difficult to detect on chest radiographs. - E5: If in the classification, no pneumonia is mild, CT may be recommended for classification. Because the ground-glass shadow and thin-film shadow cannot be seen on the chest radiography. So the diagnosis and differential diagnosis of chest radiography results may affect the diagnosis and differentiation. The severity evaluation indicators are all clinical manifestations. The chest radiography results do not affect the severity, but only the diagnosis and differentiation.   **R2:** We have revised our recommendation based on existing evidences. Studies have shown that children are more sensitive to radiation. Under the circumstances that most cases of childhood COVID-19 are mild and the relevant evidence is insufficient, it is unclear whether the use of CT for children have more benefits than harm. Therefore, CT examinations for children should be avoided as much as possible.  **Q3:** On the problem of“insufficient literature on adult imaging diagnosis”.   - E1:insufficient literature on adult imaging diagnosis.   **R3:** We have revised our inclusion and exclusion criteria and added supplementary search.  **Q4:** On the problem of“Inappropriate expression in Rationale”.   - E1:There’s inappropriate expression in Rationale , “We do not recommend CT as routine examination for children, because it has more disadvantages than advantages”. I suggest revising as “At present, there is insufficient evidence for the diagnosis of children with COVID-19, and we do not recommend routine CT examinations for the diagnosis of children.”   **R4:** We have revised our rationale as“After balance of advantages and disadvantages of radiological examination, panelists suggest do not use imaging test as routine examination for children with COVID-19”.  **Q5:** On the problem of“Lacking evidences for disadvantages of CT”.   - E1: should we explain the disadvantages of CT?   **R5:** We have revised our rationale and added related evidences.  **Q6:** On the problem of“Statement of recommendation ”   - E1: The recommendation could be formulated more carefully, e.g. that X-ray should be used only if the results of other tests were indeterminate; and CT scan never. - E2: I think ‘recommend’ should be changed to ‘suggest’ - E3: replace " recommend" with "suggest" - E4: I suggest to clarify that X-Ray is preferred for mild cases   **R6:** Based on experts’ suggestion, we have revised our recommendation and added supplementary search. Refer to the Delphi survey for specific recommendations | |

| **Clinical question 4: Should antiviral drugs such as ribavirin, interferon, remdesivir (GS-5734), lopinavir/ritonavir or oseltamivir be used to treat children with COVID-19?** | |
| --- | --- |
| **Preliminary recommendation:** | **Level of Agreement** |
| - **We recommend against using antiviral drugs for children with COVID-19. Specific antiviral drugs may be**   **administered only in the context of clinical trial. (1C)** | **Agree: 31 (94%)**  **Disagree: 0 (0%)**  **Unsure: 2 (6%)** |
| **Panelists Comments and Reply**  **This clinical question has received 9 comments from 7 panelists, our reply to the feedback is as follows:**  **Q1:** On the problem of“strength and wording of recommendations”.   - E1：We suggest against using antiviral drugs for children with COVID-19. Specific antiviral drugs may be administered only in the context of clinical trial (2C)   **R1:** We haven't revised our recommendation after weighing the risks and benefits of antiviral drugs for children with COVID-19. Refer to the Delphi survey for specific recommendations.  **Q2:** On the problem of“the uniform format of reference document”   - E2: the format of reference was uniform.   **R2:** We have revised the references form according to the periodical format. Refer to the Delphi survey for specific recommendations.  **Q3:** On the problem of“unclear description in rationale”.   - E3：Antiviral drugs are etiological treatment against viruses. Above sentence is unclear.   **R3:** We have revised our rationale and changed “Antiviral drugs are etiological treatment against viruses” into “Antiviral drugs are effective treatment against viruses which can affect the replication cycle of the virus and get them eliminated”. Refer to the Delphi survey for specific recommendations.  **Q4:** On the problems of “ recommendation of chloroquine phosphate”   - E4：The National COVID-19 Diagnosis and Treatment Guidance (sixth edition) recommended chloroquine phosphate for treatment. However, the recommended treatment dosage is large and might research rapid lethal dose easily. Close observation of adverse reactions is needed. Use of chloroquine phosphate among children is not recommended at all as child dose is more difficult to define.   **R4:** We haven't revised our recommendation. Considering the lack of available evidence for the use of chloroquine phosphate in COVID-19 population, it was only recommended to be used for the treatment of COVID-19 children under the conditions of clinical trials, but not for clinical use.  **Q5:** On the problems of “ types of drugs and related evidence”   - E1：Remdesivir (GS-5734) and chloroquine phosphate are the same drug? - E2：There is no evidence about remdesivir (GS-5734)?   **R5:** We haven't revised our recommendation. Remdesivir and chloroquine phosphate are two different drugs, and the clinical questions just listed the names of some antiviral drugs as example. There is no direct evidence of efficacy or safety of Remdesivir in COVID-19 patients.  **Q6:** On the problem of “Whether medication are recommended for children and whether to update recommendations”   - E1：ed guidelines, case reports and indirect evidence indicate that there is no effective antiviral therapy for children with COVID-19” this sentence, I checked the guidelines and consensus, found that some of them suggested use antiviral therapy for children with COVID-19. So, should we revise it? - E2：Besides, I think our recommendations should be conservative, if new clinical studies on novel coronavirus to support the use of antiviral therapy in children, should we update?   **R6:** We haven't revised this recommendation. Based on the existing suggestions for diagnosis and treatment, adults with COVID-19 can be treated with antiviral drugs such as interferon, lopinavir/litonavir or chloroquine phosphate as a try. Although the adult treatment provide lessons for the treatment of children, it is not a specific drug. Besides, indirect evidence suggests that antiviral drugs do more harm than good in COVID-19 patients, so no further modification has been made.  **Q7:** On the problem of “more indirect evidence from SARS/MERS adults was cited”   - E1: Can we quote too much evidence from SARS/MERS adult? Most adults in Beijing think this disease and SARS or MERS are not the same. We can cite the current paediatric published on the treatment of this disease, and if we can cite the mechanism of these drugs and their side effects in pediatrics for theory supportment?   **R7:** We haven't revised this part. The inclusion criteria for this rapid review were clinical trials or observational studies on the efficacy and safety of antiviral drugs, and the literature of basic studies were not included because they had not been tested in humans. Although the clinical evidence of SARS/MERS in adults is indirect, it can still provide certain reference because it has been studied in human bodies. | |

| **Clinical question 5: Should systemic corticosteroids be used to treat children with severe COVID-19?** | |
| --- | --- |
| **Preliminary recommendation:** | **Level of Agreement** |
| - **We recommend against using systemic glucocorticoids for children with COVID-19 routinely (1C).** - **We suggest a low dose and a short duration for severe COVID-19 children only when over inflammatory reaction or in the context of clinical trials. (2D)** | **Agree: 26 (79%)**  **Disagree: 3 (9%)**  **Unsure: 4 (121%)** |
| **Panelists Comments and Reply**  **This clinical question has received 12 comments from 12 panelists, our reply to the feedback is as follows:**  **Q1:** On the problem of “Changes in the wording of the recommendations section”.   - E1: replace " recommend" with “suggest” - E2：I think recommend should be replaced with suggest   **R1:** We have revised our recommendation. Refer to the Delphi survey for specific recommendations.  **Q2:** On the problem of “The timing and type of recommended systemic glucocorticoid dose”.   - E1：Steroids have to be used with great care. The guideline should clarify that the general indications for use of steroids in respiratory conditions such as ARDS may be applicable to COVID-19 also, although this has not been proven. However, cases without ARDS may not require treatment with steroids. Further, a clear note of dose, duration and type of steroid to be used should be mentioned - E2：It is very hard to decide where and when to use steroid even after carefully weighing the advantages and disadvantages.   **R2:** We haven't revised our recommendation. After careful analysis and consideration of the opinions of the above experts, our research group reviewed the evidence again. At present, there is no reliable evidence on the dosage timing and type of glucocorticoids used in children with severe COVID-19. So only the dose and usage proposed in relevant studies are presented in the evidence summary part.  **Q3:** On the problem of “The lack of adequate evidence and reports of hormonal side effects”.   - E1：There is insufficient evidence and no side effects of hormone use have been reported.   **R3:** We have revised our recommendation and described the adverse effects of hormones in the rationale part.  **Q4:** On the problem of “no high level of evidence for efficacy and increase indirect evidence in children with SARS/MERS”.   - E1：If we recommend systemic glucocorticoids, there must be very high level of evidence confirming the efficacy of such treatment (i.e. high quality RCT in patients with COVID-19). We may want to say " Judicious use of low dose glucocorticoids may be warranted in children with severe COVID-19. - E2：Using systemic corticosteroids is controversial. How about evidence about systemic corticosteroids for children with SARS (or MERS)? I think it might provide the indirect evidence. The current evidence summary could be supplemented by indirect evidence (children with similar diseases).   **R4:** We haven't revised our recommendation. After careful analysis and full consideration of experts' opinions, our research group made a recommendation for the treatment of COVID-19 for severe children based on the RCT evidence of severe or refractory pneumonia in adults/children.  **Q5:** On the problem of “add ‘under clinical trial conditions’ to recommendations rather than rationale”.   - E1：Under clinical trial conditions" could be added to the recommendation, since it's in the Rationale.   **R5:** We have revised our recommendation. Refer to the Delphi survey for specific recommendations.  **Q6:** On the problem of “systemic glucocorticoids that may cause delayed viral clearance”.   - E1：Although low dose and short-term of systemic glucocorticoid showed may have benefits in reducing some symptoms, it may delay the virus clearance. It should be also carefully weighed in the severe cases with bacterial co-infection.   **R6:** We haven't revised our recommendation. There is no direct evidence of systemic glucocorticoid therapy in severe COVID-19 children. Indirect evidence based on SARS can only prove that the use of large doses of systemic glucocorticoids in adults may delay the clearance of the virus, while it is unknown whether small doses of hormones can delay the clearance of the virus in SARS population. Therefore, it is not stated in the recommendation specification.  **Q7:** On the problem of “The use of systemic glucocorticoids has not been shown to be effective and is not recommended”.   - E1：Based on the reviews done in cases on MERS which is closer to COVID than mycoplasma pneumoniae, retrospective observational study in which 159 out of 309 patients critically ill with MERS were given corticosteroids showed that patients who were given corticosteroids were more likely to require mechanical ventilation, vasopressors, and renal replacement therapy. In addition administration of corticosteroids was not associated with a difference in 90-day mortality (adjusted odds ratio 0.8, 95% CI 0.5–1·1; P = 0.12) but was associated with delayed clearance of viral RNA from respiratory tract secretions (adjusted hazard ratio 0.4, 95% CI 0.2–0·7; P = 0·0005).”This was also true in influenza patients where a systematic in 2019 and meta-analysis observed increased length of stay in intensive care and even the rate of secondary bacterial or fungal infections in patients who were given corticosteroids. - Clark D Russell, Jonathan E Millar J Kenneth Baillie. Clinical evidence does not support corticosteroid treatment for 2019-nCoV lung injury. S0140-6736(20)30317-2. <https://doi.org/10.1016/> 。Therefore corticosteroid treatment should not be used for the treatment of COVID-19-induced lung injury or shock outside of a clinical trial. This is the current recommendation from our interim guidance. No data from this review shows any benefit. - E2：I could not agree with the “positive” recommendation of corticosteroids use base on this summary. We should stick on the evidence from the past.<https://www.thelancet.com/journals/lancet/article/PIIS014-6736(20)30317-2/fulltext>。I do not have enough confidence that the benefit outweights the harm. - E3：Must emphasize that routine use is not recommended   **R7:** We have revised our recommendation as “We recommend against using systemic glucocorticoids for children with COVID-19 routinely. We suggest a low dose and a short duration for severe COVID-19 children only when over inflammatory reaction or in the context of clinical trials”. The evidence from the current retrospective study of 309 patients is insufficient to routinely recommend hormone use. First of all, the study population is at an average age of 57.8±17.2, the dose they use is high, and the group that used hormone has higher underlying diseases than the group without hormone (P = 0.001), so there's insufficient evidence for whether to use hormone in children. In addition, indirect evidence suggests that a short course of treatment at low doses may be effective in children with severe COVID-19 and may be used as appropriate if necessary. | |

| **Clinical question 6: Should intravenous immunoglobulin (IVIG) be used to treat children with severe COVID-19?** | |
| --- | --- |
| **Preliminary recommendation:** | **Level of Agreement** |
| - **We recommend against using intravenous immunoglobulin (IVIG) for children with severe COVID-19. (1B)** | **Agree:29 (88%)**  **Disagree:1 (3%)**  **Unsure:3 (9%)** |
| **Panelists Comments and Reply**  **This clinical question has received 2 comments from 2 panelists, our reply to the feedback is as follows**  **Q1:** On the problem of “Is a small dose of IVIG recommended”.   - E1: Maybe it should be said: high dose IVIG is not recommended, but what about small doses? National guideline has recommendations   **R1:** We haven’t revised our recommendation. Based on relevant recommendations of the National Health Commission’s guidelines and evidences related to the recommendations, we think there is no elaboration or relevant evidence to support using low-dose IVIG for children with COVID-19.  **Q2:** On the problem of “Is there necessity to keep this clinical question”.   - E1: Is there a justified reason to even consider IVIG for children with COVID-19? If not, the entire recommendation may not be necessary. (I'm asking because I'm not a clinician so I don't know what are the typical kinds of treatment used for respiratory diseases/viral infections)   **R2:** We haven’t deleted this clinical question. Based on the questionnaire of clinical questions in the early stage, the importance of this clinical question got high score from panelists and ranked ahead. | |

| **Clinical question 7: Should antibiotic agents be used to treat children with COVID-19?** | |
| --- | --- |
| **Preliminary recommendation:** | **Level of Agreement** |
| - **We recommend against the use of antibiotic agents for children with COVID-19 when there is no evidence of bacterial coinfection. (1B)** | **Agree: 33 (100****%)**  **Disagree: 0 (0%)**  **Unsure: 0 (0%)** |
| **Panelists Comments and Reply:**  **This clinical question has received 3 comments from 2 panelists, our reply to the feedback is as follows:**  **Q1****:** On the problem of “The term ‘Stenomonas’ should be spelled as ‘Stenotrophomonas’”.  **R1:** We have revised our rationale and evidence and changed “Stenomonas” into “Stenotrophomonas”.  **Q2:** On the problem of “‘Aspergillus flavus’ is a fungus and should not be listed in the secondary infection of COVID-19”.  **R2:** We have revised our rationale and evidence and removed “Aspergillus flavus” from the examples of secondary infection. At present, we list the main types of bacterial infection.  **Q3：**On the problem of “the sentence ‘four case series[9-12]showed that comprehensive treatment including antibiotic agents had some therapeutic effects on SARS’ is not specific enough”.  **R3:** We have revised our evidence as “four case series [9-12] showed that comprehensive treatment including antibiotic agents had some therapeutic effects in alleviating symptoms and reducing mortality in SARS patient”. | |

| **Clinical question 8：How should parents be advised to get information on SARS-CoV-2 infection?** | |
| --- | --- |
| **Preliminary recommendation:** | **Level of Agreement** |
| - **We recommend parents to obtain information regularly from academic and official resources rather than social media. (1D)** | **Agree: 31 (94%)**  **Disagree: 0 (0%)**  **Unsure: 2 (6%)** |
| **Panelists Comments and Reply:**  **This clinical question has received 14 comments from 8 panelists, our reply to the feedback is as follows:**  **Q1:** On the problem of "social media is widely used, real-time updating and paper media lags behind"   - - - - E1: Second point: Information circulating in social media may likely be unreliable, but social media is also an increasingly utilized method of communication (even among scientists). Its advantage is that the information is in real-time and can be more up to date that traditional media. The recommendation could be reformulated more softly e.g. "..., and interpret information from social media with caution"       - E2: for the second points, I think we should be careful about the types of sources of information, the social media can include the wechat/weibo account of national authorities (the official account in social media should be trusted). About print media--I am not sure how many people will read the newspaper? In addition, could we consider listing some examples of "authorities and official agencies"?       - E3：print media--I am not sure how many people will read the newspaper? In addition, could we consider listing some examples of "authorities and official agencies"       - E4: We recommend excluding print media from the second item, as print media is not fully controlled and there could be a risk of of misinformation.   **R1:** We have revised our recommendation based on existing evidences. Refer to the Delphi survey for specific recommendations.  **Q2:** On the problem of “hand washing, cough etiquette, etc”.   - E1:also give concrete advice to children on issues like washing hands, not to cough/sneeze towards other people, etc。 - E2: teach their children Basic knowledge of infectious diseases and how to properly wash hands, cough etiquette, etc. - E3: parents or family members should consider teaching children about behaviors, e.g. how to protect themselves, wash hands before eating, reducing touching faces/mouths/eyes with unwashed hands, how to cough or sneeze? how to wear mask? when to wear mask?   **R2:** We have revised our rationale and added the information in the rationale.  **Q3:** On the problem of“teach children not to discriminate people”.   - E1: delete the "and teach their children not to discriminate people from areas affected by the epidemic" The second half of the sentence has no relationship with disease prevention. - E2: for the third point, I think only awareness is not enough, parents or family members should consider teaching children about behaviors. - E3: teach their children not to discriminate people from areas affected by the epidemic, This is inappropriate, including deeper and more complex ethical issues, and is not the focus of current guidelines.   **R3:** We have revised our recommendation based on the available evidence and deleted this information.  **Q4:** On the problem of "to modify the clinical questions".   - E1: suggest minor changes in the wording of the question——"about preventing ...infection", or "about the prevention of ..infection" - E2: The question itself is problematic. If you keep the current question and ask parents how to get advice, the suggestion should include only the second recommendation. If you need to include all three, the question should be deleted “to get information”.   **R4:** We have revised the question in the Delphi survey documents.  **Q5:** On the problem of “Questions about health education, such as ‘school science popularization’ and ‘children are too young to accept’”.   - E1: Note that if children are too young, health education cannot be implemented. - E2: Students listen more to the teacher. Is it recommended that schools push some scientific knowledge on disease prevention to guide students!   **R5:** We have revised our recommendation and added the information in the rationale. | |

| **Clinical question 9: Should mothers with COVID-19 continue to breastfeed their babies?** | |
| --- | --- |
| **Preliminary recommendation:** | **Level of agreement** |
| - **We recommend against mothers interrupting breastfeeding. (2C)** | **Agree: 23 (72%)**  **Disagree: 3 (9%)**  **Unsure: 6 (19%)** |
| **Panelists Comments and Reply**  **This clinical question has received 17 comments from 15 panelists, our reply to the feedback is as follows**  **Q1:** On the problem of “suggest adding under appropriate precautions”.   - E1:We should add: However, breastfeeding mothers should take precautions to minimize exposure to their infants by frequent hand hygiene, avoidance of coughing over the infant, etc.   **R1:** We have added “Mothers should not interrupt breastfeeding under appropriate precautions, including wearing masks, hand hygiene and so on” in our rationale.  **Q2:** On the problem of“suggest expressing breast milk instead?”   - E1: To avoid close contact, do we suggest expressing breast milk instead of breastfeeding?   **R2:** We haven’t revised our recommendation to suggest all mothers to express breast milk instead. Since breastfeeding has various advantage compared with artificial feeding, we do suggest mothers trying to continue breastfeeding under appropriate precautions if possible. But considering some mothers would worry about the possibility of transmission, so We change our rationale as “Mothers should not interrupt breastfeeding under appropriate precautions, including wearing masks, hand hygiene and so on, or expressing breast milk as an alternative”.  **Q3:** On the problem of“Since COVID-19 can be transmitted via close contact and droplets, and mothers should be isolated, do we recommend interrupting breastfeeding?”   - E1:Although there is no direct evidence indicating that SARS-CoV-2 could be transmitted via breast milk，but nCOV transmission through droplets and contact during breastfeeding is possible. Both mild and severe patients are the source of infection. - E2: The close contact with mothers with COVID-19 will increase the risk of infection with baby, so the breastfeeding should be continued after the viral nucleic acid turn negative. - E3: There were little the information about breastfeeding from SARS-CoV-2 infected mother depending on reviews and case reports at present. On the other hand, the SARS-CoV-2 infected mother would transmit the virus to the baby when they breastfeed to baby by herself. By far, there have been two cases report about newborn with SARS-CoV-2 infected in Wuhan. So I don't recommend breastfeeding from SARS-CoV-2 infected mother, just in case now. - E4: The limited evidence does suggest that the newborns can get infected easily, most likely from their mothers. Due to the limited number of cases of infection in newborn, we cannot be sure that all of them will have very mild disease. If one in 30 newborns develops severe disease, do we still recommend SARS-CoV-2 infected mother continue to breastfeed their babies? What is the down-side of withholding breast feeding for two weeks? Mother can continue to pump to maintain the flow of breast milk. You may want to recommend: Newborns can get infected by contact with their mothers. Limited evidence suggested that newborns usually do not develop severe illness. Mother may want to continue breast feeding when they are no longer infectious. - E5:For the two newborn COVID-19 cases at Wuhan Children’s Hospital, the youngest baby was infected by COVID-19 only 30 hours after birth. His mother is confirmed case of COVID-19. The existence of vertical mother-to-child transmission was considered by experts. Nanshan Zhong’s team isolated SARS-CoV-2 from the mother’s urine on 22nd February , which suggested that the previous negative test results of SARS-CoV-2 of mother’s milk might be due to technical issues. Breast feeding makes close contact and can transmit virus directly or indirectly. Breast feeding is not recommended for confirmed cases of COVID-19, quarantine should be guaranteed.   **R3:** We haven’t revised our recommendation. As far as existing evidences indicate no viral nuclear acids are found in breast milk samples from seven COVID-19 mothers, there is no evidence that COVID-19 can be transmitted via breast milk. As some experts mention there have been cases of neonates infected with SARS-CoV2, but these cases are also not sure whether COVID-19 can be transmitted via breastfeeding, since there’s possibility of intrauterine vertical transmission. Existing studies have shown that the symptoms of COVID-19 mostly tend to be asymptomatic or mild in children, as far as we know there is only one severe case in children reported. So, we hold the view that the advantages of breastfeeding outweigh the risks related to the possible infection. So, we insist our recommendation as “We recommend against mothers interrupting breastfeeding”.  **Q4:** On the problem of“the strength and quality of recommendation and evidence?”   - E1: How about the quality of evidence and strength of recommendation?   **R4:** We have revised our recommendation and added the information. The quality of evidence and strength of recommendation is 2C. | |

| **Clinical question 10: What are the appropriate means of supportive care for children with severe COVID-19?** | |
| --- | --- |
| **Preliminary recommendation:** | **Level of Agreement** |
| **We propose the following forms of supportive care for children with severe COVID-19:**  **1)ensuring sufficient number of adequate medical staff;**  **2)systematically monitoring and recording vital signs;**  **3)using supportive care of the respiratory and cardiovascular symptoms according to clinical needs;**  **4)providing psychological interventions.** | **Agree 32(97%) Disagree 1(3 %) Unsure 0 (0%)**  **Agree 33(100%) Disagree 0 (0%) Unsure 0 (0%)**  **Agree 33(100%) Disagree 0 (0%) Unsure 0 (0%)**  **Agree 29(88%) Disagree 1(3%) Unsure 3(9%)** |
| **Panelists Comments and Reply**  **This clinical question has received 9 comments from 9 panelists, our reply to the feedback is as follows**  **Q1:** On the problem of“ensuring sufficient number of adequate medical staff in ICUs”   - E1: These are good and important points, but their feasibility depends strongly on the resources. The guidelines should also consider the situation where the virus starts to spread in resource-limited settings. Setting too high requirements may in worst case become counterproductive and stop children getting any care. An addition about what to do e.g. in countries and settings where most people cannot access a hospital with ICU could be useful. - E2: Delete "in ICUs". Not only ICU should be fully staffed, but other medical departments should also be adequately staffed. - E3: Replace “ICU” with “PICU”。 - E4: We should either specify what we mean by adequate number of staff or delete this. Further, what is the evidence that having staff will help, if the material resources (ICU beds, protective equipment etc) are lacking.   **R1:** We have deleted “ICU”. Because the medical service of each country is different, the access of ICU support is also not identical. So, we no longer stress that ICU should be equipped with plenty of medical staff, but emphasize that during the period of COVID-19 outbreak, the whole medical system, from each unit in the hospital was able to be equipped with adequate medical staff to work together to provide patients with treatment.  **Q2:** On the problem of “using supportive care of the respiratory and cardiovascular system according to clinical needs”   - E1: The recommendation should have been more specific, especially early use supplemental oxygen therapy in cases of hypoxia.   **R2:** We haven’t revised. We believe that even suffering the same disease, patients show different response to infection or therapy. We can only recommend that according to the specific clinical situation, give patients appropriate respiratory and circulatory support, while specific details of respiratory and circulatory support cannot be given.  **Q3:** On the problem of“providing psychological therapy for children with severe COVID-19”   - E1: Family support should be suggested, since children are so young and family concerns may be more beneficial when childern were treated in isolation ward,. In addition, counselling may be difficult to be comprehended and counselling for family members may be more useful. - E2: The visits from their parents or family members should be considered. - E3: Psychological intervention is also applicable to the parents of children with severe COVID-19. In addition, psychological therapy is only suitable for children over school age. - E4: Psychological support should give to all sick kids with COVID-19 and to all kids with family members suffering from COVID-19, esp death.   **R3:** We have revised and replaced “therapy” with “intervention”. It was emphasized that on the basis of “patient-centered”, communication between medical staff and children can help relieve the anxiety of children, rather than professional psychotherapy or intervention. Supplementary data about the importance of psychological counseling for parents were shown in the evidence. | |

**Basic Terminology**

| **No** | **Terms** | **Initial definition** |
| --- | --- | --- |
| 1 | Severe Acute Respiratory Syndrome Coronavirus 2 (SARS-CoV-2) | A novel coronavirus (CoV) is a new strain of coronavirus that has not been previously identified in humans. The virus that caused the outbreak of respiratory illness first detected in Wuhan, China, is named Severe Acute Respiratory Syndrome Coronavirus 2 (SARS-CoV-2) [1]. This is an enveloped RNA virus belonging to the β Genus, with a diameter of 60-140nm [2]. The genetic characteristics of SARS-CoV-2 are different from those of the severe acute respiratory syndrome coronavirus (SARS-CoV) and the Middle East respiratory syndrome coronavirus (MERS-CoV). The genome of SARS-CoV-2 may be more than 85% identical with the genome of a bat SARS-like CoV (bat-SL-CoVZC45, MG772933.1) [2]. The official name given by the World Health Organization to the disease caused by SARS-CoV-2 is Coronavirus Disease 2019 (COVID-19).[3]  Ref:  1.CDC. Coronavirus Disease 2019 (COVID-19) Situation Summary. 2020. https://www.cdc.gov/coronavirus/2019-nCoV/summary.html (accessed Feb 16, 2020)  2.New coronavirus pneumonia prevention and control program (6nd ed.) (in Chinese). 2020 http://www.nhc.gov.cn/yzygj/s7653p/202002/8334a8326dd94d329df351d7da8aefc2/files/b218cfeb1bc54639af227f922bf6b817.pdf.  3. WHO. Naming the coronavirus disease (COVID-2019) and the virus that causes it. 2020.https://www.who.int/emergencies/diseases/novel-coronavirus-2019/technical-guidance/naming-the-coronavirus-disease-(covid-2019)-and-the-virus-that-causes-it |
| **Questions and answers：**  **Q1：**I suggest use nCoV-2019 as the name of the virus, instead of SARS-CoV-2  **R1:** We haven’t revised because the official name of the virus is SARS-CoV-2 according to WHO and CDC. (Ref: WHO <https://www.who.int/emergencies/diseases/novel-coronavirus-2019/technical-guidance/naming-the-coronavirus-disease-(covid-2019)-and-the-virus-that-causes-it>. CDC：https://www.cdc.gov/coronavirus/2019-ncov/summary.html。Q2：Grammatical mistakes  **Q2：**The genome of SARS-CoV-2 may however be more than 85% identical with the genome of a bat SARS-like CoV. Delete: however  **R2:** We have revised as you suggest. | | |
| 2 | Incubation Period | Incubation period refers to the interval between exposure to COVID-19 and the symptom onset. Incubation period of COVID-19 is usually 1 to 14 days, and in most cases between 3 and 7 days [1,2]. Incubation period is observed to be 24 days in one study. [3].  Ref:  1.Li Q, Guan X, Wu P, et al. Early Transmission Dynamics in Wuhan, China, of Novel Coronavirus-Infected Pneumonia [published online ahead of print, 2020 Jan 29]. N Engl J Med. 2020;10.1056/NEJMoa2001316. doi:10.1056/NEJMoa2001316  2.New coronavirus pneumonia prevention and control program (6nd ed.) (in Chinese). 2020 <http://www.nhc.gov.cn/yzygj/s7653p/202002/8334a8326dd94d329df351d7da8aefc2/files/b218cfeb1bc54639af227f922bf6b817.pdf.>  3.Guan W-j, Ni Z-y, Hu Y, Liang W-h, Ou C-q, He J-x, et al. Clinical characteristics of 2019 novel coronavirus infection in China. medRxiv. 2020:2020.02.06.20020974. doi: 10.1101/2020.02.06.20020974. |
| **Questions and answers：**  **Q1:** The above description is about the incubation period of adults. Should children's incubation period be defined?"2)Show IP separately for adults and children even if latter is from unpublished data  **R1:** Thanks for your suggestions. One unpublished study has reported children's incubation period. We will add children's incubation period in the definition after the study published.  **Q2:** 1)Start with: Incubation period of COVID-19 refers to the interval between … 2) should we consider explaining what is "incubation period" (how to define it) 3) Since this document is to explain the terminology, a brief formal definition of the term could be added (time from infection to onset of symptoms)  **R2：**We have added the definition of the term.  **Q3:** even longer?  **R3:**Thanks for your suggestion. No evidence has shown a longer period.  Q4:1)This sentence "The longest incubation period observed is 24 days" could be revised to " there is very little that incubation period observed is 24 days" .otherwise ,we will be confused.2)Limited evidence showed the longest incubation period observed was 24d.  R4: We haven’t revised. Please refer to the terminology for details. | | |
| 3 | Route and Mode of Transmission | The main route of the transmission of SARS-CoV-2 is through respiratory droplets and close contact [1,2]. Transmission may occur through aerosol when exposed to high concentrations of aerosols in a relatively closed environment for a long time [2] and oral-faecal route [3]. Whether transmission can occur through urinary tract, or from mother to infant either in utero [4], perinatally or through breast milk, has not been established yet.  Ref:  1.CDC. How COVID-19 Spreads. 2020. https://www.cdc.gov/coronavirus/2019-ncov/about/transmission.html (accessed Feb 16, 2020)  2.New coronavirus pneumonia prevention and control program (6nd ed.) (in Chinese). 2020 <http://www.nhc.gov.cn/yzygj/s7653p/202002/8334a8326dd94d329df351d7da8aefc2/files/b218cfeb1bc54639af227f922bf6b817.pdf.>  3.Xiao F, Tang M, Zheng X, Li C, He J, Hong Z, et al. Evidence for gastrointestinal infection of SARS-CoV-2. medRxiv. 2020:2020.02.17.20023721. doi: 10.1101/2020.02.17.20023721.  4.Chen H, Guo J, Wang C, et al. Clinical characteristics and intrauterine vertical transmission potential of COVID-19 infection in nine pregnant women: a retrospective review of medical records. The Lancet, 2020,DOI:https://doi.org/10.1016/S0140-6736(20)30360-3. |
| **Questions and answers：**  **Q1：**1) I think feco-oral transmission has been recently documented; 2) fecal-oral transmission may also exist  **R1:** Thanks for your suggestions. We have added the feco-oral transmission in the definition.  **Q2：**Please add：Whether transmission can occur through urinary tract has not been established yet.  **R2：**Thanks for your suggestions. We have added it.  **Q3：**Change “close contact” to “contact transmission” (contact transmission includes close contact )。  **R3：**Thanks for your suggestions. We haven’t revised because “Close contact” is one of the main transmission mode based on the evidence in “New coronavirus pneumonia prevention and control program (6nd ed.) (in Chinese). 2020”.  **Q4：**Should we consider explaining what is "close contact" (hug? face-to-face eating? shake hands? kiss? sexual contact? how to define it) ? --after this comment, I saw term 5 had the definition and should we change the order or refer guideline users/readers to go to term  **R4：**Thanks for your suggestions. The definition of close contact is detailed in terminology 5, and will be presented in a way that is easy for readers to identify, such as an index.  **Q5:** Transmission route through Direct contact (deposited on persons) or Indirect contact (deposited on objects) should also be mentioned.  **R5:** Thanks for your suggestions. Close contacts include direct contact and indirect contact.  **Q6:** Should we give negative evidence for mother to infant transmission?  **R6：**Thanks for your suggestions. We have added related reference. | | |
| 4 | History of Epidemic Exposure (Contact) | Epidemic exposure to SARS-CoV-2 is defined as having at least one of the following[1, 2]:  • A history of travel to or residence in the city of Wuhan (Hubei Province, China), or other communities where cases have been reported within 14 days.  • Close contact with patients with fever or respiratory symptoms who came from the city of Wuhan (Hubei Province, China) or communities where cases have been reported within 14 days.  • Close contact with a confirmed case of COVID-19 within 14 days.  • Close contact with a cluster outbreak.  There is as yet, no separate criteria for children.  Ref:  1.WHO. Clinical management of severe acute respiratory infection when novel coronavirus (nCoV) infection is suspected. Jan 11, 2020. https://www.who.int/internal-publications-detail/clinicalmanagement-of-severe-acute-respiratory-infection-when-novelcoronavirus-(ncov)-infection-is-suspected (accessed Jan 19, 2020)  2.CDC. Coronavirus Disease 2019 (COVID-19) Situation Summary. 2020. https://www.cdc.gov/coronavirus/2019-nCoV/summary.html (accessed Feb 16, 2020) |
| **Questions and answers：**  **Q1:** Please add that there is as yet, no separate criteria for children.  **R1：**Thanks for your suggestion. We have added that in the definition.  **Q2：**As mentioned before, the longest incubation periods is 24 days. Is it accurate to define “with 14 days”?  **R2：**Thanks for your suggestion. We have modified the definition of incubation period to avoid confusion.  **Q3:** 1)As we know some people might be asymptomatic communicator, so “14 days prior to symptom onset.” or “14 days prior to illness onset” is not appropriate , I recommend this two sentence change to “Close contact with people from the city of Wuhan (Hubei Province, China), or patients with fever or respiratory symptoms who came from communities where cases have been reported within 14 days”, “Close contact with a confirmed case of COVID-19 within 14 days. 2)14 days should always be "14 days or less"  **R3：**Thanks for your suggestions. We have changed "14 days prior to symptom onset" into "within 14 days".  **Q4：**”people” should be modified to another word.  **R4：**Thanks for your suggestions. We have changed “people” into “patients with fever or respiratory symptoms”.  **Q5：**Unify the form of references  **R5：**Thanks for your suggestion. We have revised the form of reference.  **Q6:** "Connection" (to a cluster) could be defined more specifically  **R6：**Thanks for your suggestions. We have changed "Connection" into "Close contact with".  **Q7:** now it's not just Wuhan  **R7:** Thanks for your suggestions. Probable cases of COVID-19 who is not in Wuhan are in the category of “other communities where cases have been reported”. | | |
| 5 | Close Contact | Close contact is defined as a) being within approximately 2 meters (6 feet) of a COVID-19 case for a prolonged period of time. Close contact can occur while living with, visiting, or sharing a health care waiting area or room with a COVID-19 case or b) having direct contact with infectious secretions of a COVID-19 case (e.g., being coughed on) [1].  Ref:  1.CDC. Recommendations for Reporting, Testing, and Specimen Collection.https://www.cdc.gov/coronavirus/2019-ncov/hcp/clinical-criteria.html?CDC_AA_refVal=https%3A%2F%2Fwww.cdc.gov%2Fcoronavirus%2F2019-ncov%2Fclinical-criteria.html (accessed Feb 3, 2020) |
| **Questions and answers：**  **Q1:** Close contact can occur in Public transport?  **R1:** Thanks for your suggestion, but we haven’t revised because at present we haven’t found any reported studies related.  **Q2:** Change “close contact” to “contact transmission” (contact transmission includes close contact ).  **R2：**Thanks for your suggestions. We haven’t revised because “Close contact” is one of the main transmission mode based on the evidence in “New coronavirus pneumonia prevention and control program (6nd ed.) (in Chinese). 2020”.  **Q3:** Add: with or without protection?  **R3:** Thanks for your suggestion. but we haven’t revised because at present we haven’t found any reported studies related.  **Q4：**Modify the form of references  **R4：**Thanks for your suggestion. We have modified them.  **Q5:** a prolonged period of time？Delete of give a specific time.  **R5:** Thanks for your suggestion. But we haven’t revised because at present we haven’t found any reported studies showing a specific time. | | |
| 6 | Suspected Case (Person Under Investigation) | Children with any acute viral respiratory illness (fever and/or respiratory symptoms) [1, 2]or other symptoms such as gastrointestinal symptoms (vomiting and/or diarrhoea),or asymptomatic children with abnormal chest CT [3],with a history of epidemic exposure as mentioned above.  Ref:  1.WHO. Global Surveillance for human infection with novelcoronavirus (2019-nCoV)Interim guidance. Jan 31, 2020.<https://www.who.int/publications-detail/global-surveillance-for-human-infection-with-novel-coronavirus-(2019-ncov)>  2.CDC. Evaluating and Reporting Persons Under Investigation (PUI). 2020. <https://www.cdc.gov/coronavirus/2019-ncov/hcp/clinical-criteria.html?CDC_AA_refVal=https%3A%2F%2Fwww.cdc.gov%2Fcoronavirus%2F2019-ncov%2Fclinical-criteria.html>  3. Chan JF, Yuan S, Kok KH, et al. A familial cluster of pneumonia associated with the 2019 novel coronavirus indicating person-to-person transmission: a study of a family cluster. Lancet. 2020;395(10223):514–523. doi:10.1016/S0140-6736(20)30154-9 |
| **Questions and answers：**  **Q1：**Asymptomatic children have a close contact with a cluster outbreak, should we consider these children as suspected case？  **R1：**Thanks for your suggestion. We haven’t revised because we can’t make sure weather these children are suspected cases. They need watchful isolation at home for 14 days. If any symptoms occur, they could be treated as suspected cases.  **Q2:** 1)Please note that the symptoms in children may not necessarily confined to the respiratory system. Therefore, children with other symptoms such as gastrointestinal symptoms (vomiting and diarrhoea) with a history of exposure should also be investigated. 2)what about fever and/or gastrointerstinal synmtoms or others, or abnormal chest CT?  **R2：**Thanks for your suggestion. We have added gastrointestinal symptoms and abnormal chest CT in the definition.  **Q3:** Should we add pathogenic evidence in the definition.  **R3:** Thanks for your suggestion. We have added pathogenic evidence in the definition of confirmed case.  **Q4:** Should we indicate "children" in the definitions?  **R4：**Thanks for your suggestion. We have edited it. | | |
| 7 | Confirmed Case | Suspected children with at least one of the following types of pathogenic evidence[1, 2]:  (1) positive result for SARS-CoV-2 by Reverse Transcription-Polymerase Chain Reaction(RT-PCR) test for nucleic acid in respiratory or blood or stool samples.  (2) viral gene sequencing showing high homegeneity to SARS-CoV-2 in respiratory or blood or stool samples.  Ref:  1.WHO. Global Surveillance for human infection with novelcoronavirus (2019-nCoV)Interim guidance. Jan 31, 2020.<https://www.who.int/publications-detail/global-surveillance-for-human-infection-with-novel-coronavirus-(2019-ncov)>  2.New coronavirus pneumonia prevention and control program (6nd ed.) (in Chinese). 2020 (http://www.nhc.gov.cn/yzygj/s7653p/202002/8334a8326dd94d329df351d7da8aefc2/files/b218cfeb1bc54639af227f922bf6b817.pdf). |
| **Questions and answers：**  **Q1:** "Patient" change into "Suspected patient"  **R1：**Thanks for your suggestion. We have edited it.  **Q2:** 1) Add: stool specimen；2) Patients with stool samples or rectal swabs positive should also be considered as confirmed cases.3)  **R2：**Thanks for your suggestions. We have added it.  **Q3:** Should explain "RT-PCR"  **R3：**Thanks for your suggestions. We have explained as "Reverse Transcription-Polymerase Chain Reaction".  **Q4:** Should we add time limitation?  **R4:** Thanks for your suggestion. but we haven’t revised because at present we haven’t found any reported studies related.  **Q5:** Should we considered the new diagnosis criteria in Hubei province, such as clinical indications, the CT imaging results?  **R5 ：**Thanks for your suggestions. We haven’t revised because we do not recommend CT for diagnosis of children with COVID-19. | | |
| 8 | Asymptomatic Infection | Children who have been exposed to a confirmed or probable case of COVID-19[1], and are tested positive for SARS-CoV-2 without manifestations of any clinical symptoms or abnormal chest imaging findings[2].  Ref:  1. WHO. Global Surveillance for human infection with novelcoronavirus (2019-nCoV)Interim guidance. Jan 31, 2020.<https://www.who.int/publications-detail/global-surveillance-for-human-infection-with-novel-coronavirus-(2019-ncov)>  2. Shen K, Yang Y, Wang T, et al. Diagnosis, treatment, and prevention of 2019 novel coronavirus infection in children: experts' consensus statement [published online ahead of print, 2020 Feb 7]. World J Pediatr. 2020;10.1007/s12519-020-00343-7. doi:10.1007/s12519-020-00343-7 |
| **Questions and answers：**  **Q1:** Clearly indicate a SARS-CoV-2 positive nucleic acid test  **R1:** Thanks for your suggestions. Pathogenic evidence includes RT-PCR test or viral gene sequencing. So "tested positive for SARS-CoV-2" may be better.  **Q2:**"Infection in children who have been in contact with a confirmed or probable case of COVID-19" change into "Children who have been in contact with a confirmed or probable case of COVID-19"  **R2：**Thanks for your suggestions. We have modified it.  **Q3:** Change "contact" to "expose"  **R3：**Thanks for your suggestions. We have modified it.  **Q4：**How about children who have positive result for SARS-CoV-2 without contact history?  **R4:** Thanks for your suggestions. We haven’t revised because we think children without epidemic exposure to SARS-CoV-2 don’t need to detect SARS-CoV-2. Because existing studies of this kind of case are very rare. | | |
| 9 | Mild Case | Children with a SARS-CoV-2 infection in the upper respiratory tract (fever, cough and/or fatigue et al), without manifestation of pneumonia [1, 2].  Ref:  1.WHO. Clinical management of severe acute respiratory infection when novel coronavirus (nCoV) infection is suspected. Jan 11, 2020. https://www.who.int/internal-publications-detail/clinicalmanagement-of-severe-acute-respiratory-infection-when-novelcoronavirus-(ncov)-infection-is-suspected (accessed Jan 19, 2020)  2.Shen K, Yang Y, Wang T, et al. Diagnosis, treatment, and prevention of 2019 novel coronavirus infection in children: experts' consensus statement [published online ahead of print, 2020 Feb 7]. World J Pediatr. 2020;10.1007/s12519-020-00343-7. doi:10.1007/s12519-020-00343-7 |
| **Questions and answers：**  **Q1:** The main symptoms should be listed as fever, cough and fatigue, and the upper respiratory tract infection is too vague  **R1:** Thanks for your suggestions. We have modified it.  **Q2:** In Term 10, we said severe pneumonia, how about mild pneumonia--it is mild case or severe case?  **R2:** Thanks for your suggestions. We haven’t revised because the severity classification is based on SARSCoV-2 infection, not pneumonia. | | |
| 10 | Severe Case | Children with COVID-19 presenting the criteria of severe pneumonia (such as breathing faster, dyspnea, respiratory distress, hypoxemia, change of consciousness, or trouble feeding) and critical illness (such as respiratory failure, septic shock or other organ failure requiring intensive care)[1,2].  Ref:  1.WHO. Clinical management of severe acute respiratory infection when novel coronavirus (nCoV) infection is suspected. Jan 11, 2020. https://www.who.int/internal-publications-detail/clinicalmanagement-of-severe-acute-respiratory-infection-when-novelcoronavirus-(ncov)-infection-is-suspected (accessed Jan 19, 2020)  2.Pocket book of hospital care for children: Guidelines for the management of common childhood illnesses  [http://www.who.int/maternal_child_adolescent/documents/child_hospital_care/en/]. 2nd ed. Geneva: WHO; 2013.  3.Shen K, Yang Y, Wang T, et al. Diagnosis, treatment, and prevention of 2019 novel coronavirus infection in children: experts' consensus statement [published online ahead of print, 2020 Feb 7]. World J Pediatr. 2020;10.1007/s12519-020-00343-7. doi:10.1007/s12519-020-00343-7 |
| **Questions and answers:**  **Q1:**Added breathing faster, dyspnea before respiratory distress, because these terms describe breathing differently.  **R1:** Thanks for your suggestions. We have added it. | | |
| 11 | Self-Isolation | If children are asked to self-isolate, they should stay home and avoid going to a public area and attending social gatherings until 14 days after the date of their suspected epidemic contact or exposure to SARS-CoV-2. In addition, their caregivers have to report any symptoms of COVID-19 immediately to their health providers. Appropriate personal protective equipment and hygiene practices should be used when close contact is needed.  Ref:   1. CDC. Interim Guidance for Preventing the Spread of Coronavirus Disease 2019 (COVID-19) in Homes and Residential Communities.https://www.cdc.gov/coronavirus/2019-ncov/hcp/guidance-prevent-spread.html (accessed Feb 20, 2020) 2. WHO. Home care for patients with suspected novel coronavirus (nCoV) infection presenting with mild symptoms and management of contacts. Feb4, 2020.https://www.who.int/publications-detail/home-care-for-patients-with-suspected-novel-coronavirus-(ncov)-infection-presenting-with-mild-symptoms-and-management-of-contacts (accessed Jan 19, 2020) |
| **Questions and answers: None** | | |

**Pathway**

| **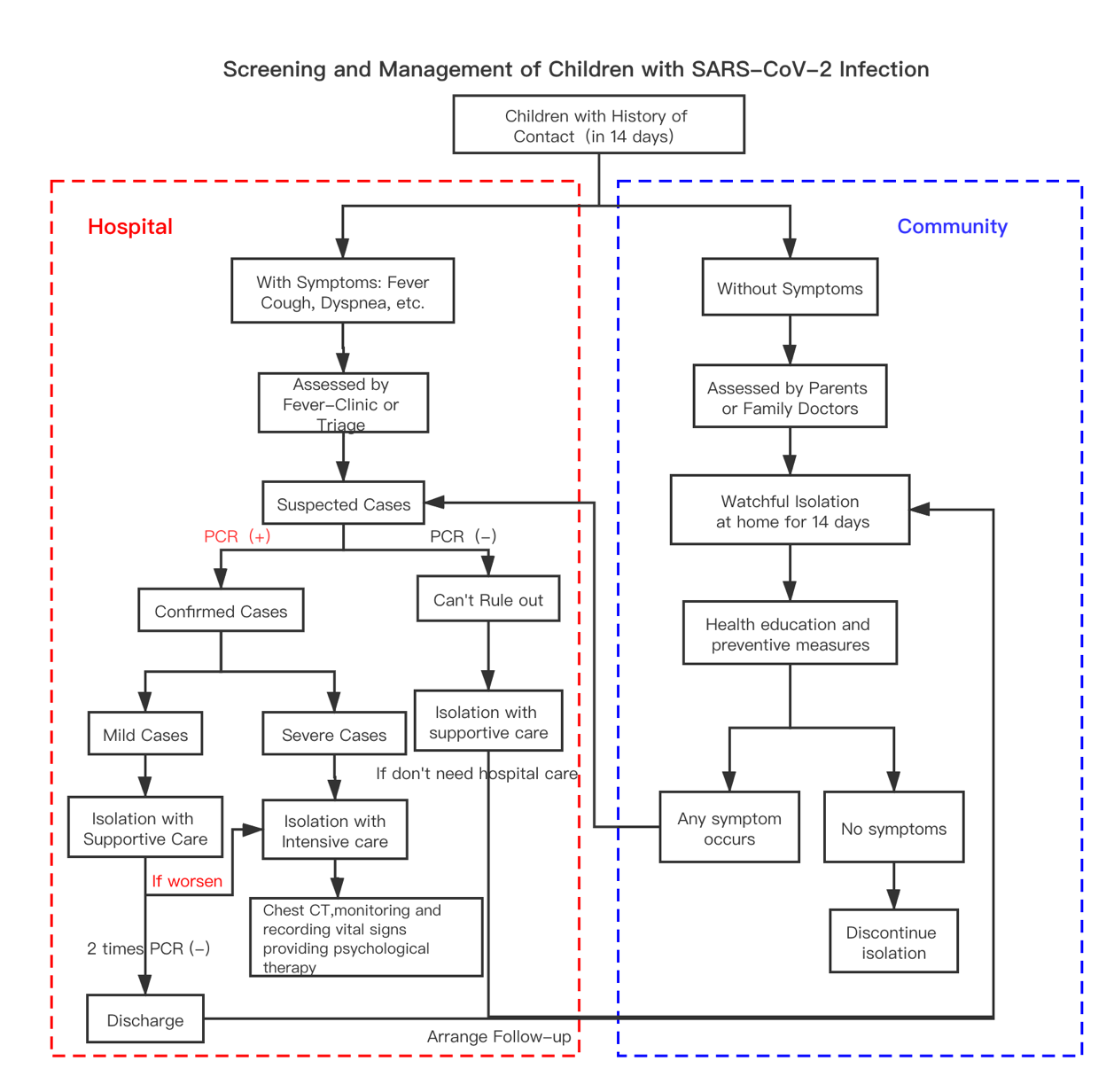** |
| --- |
| **Q1:** Title   - E1:Change title as ”Screening and management of Children with SARS-Cov-2”. - E2: The title of the pathway is for "children with COVID-19, HOWEVER, the first box is for children with history of contact   **R1:** Thanks for your suggestions. We have modified it.  **Q2:** Next step after chest CT in mild case   - E1: Only need CT screening when mild case aggravate? - E2: what is next step after chest CT? - E3: also for severe cases should be noted what happends after chest CT.   **R2:** We have add CT and supportive treatment.  **Q3:** Add PCR or CT for asymptomatic person   - E1: An asymptomatic person should be given a nucleic acid test if possible. - E2: For those who can't be excluded by PCR (PCR negative) for the first time, conduct additional examination with low dose CT. If CT result shows signs of viral pneumonia, implement quarantine and conduct PCR for a second time.   **R3:** No modification has been made after discussion in order to keep the flow chart consistent with the recommendation of the guide.  **Q4:** Negative PCR (can't rule out)still unclear: is it also 14 days;  **R4:** We are trying to make it clear. Add the follow-up. |

**Other questions:**

**Q1:** Should we consider adding "Abbreviations and acronyms"?

**R1:** Thanks for your suggestions. We have added them.

**Q2:** how to rank these terms?

**R2:** Thanks for your suggestions. We will rank these terms according to the order of appearance in full text.

**Panelists Comments Feedback Report on Second Round Recommendations Survey**

**Notes:** As far to March 4th, 2020, the second-round survey of recommendations on the guidelines had collected questionnaire results from 30 panelists. The survey results and their feedback have been added in the following table.

1. The comments of panelists have been replied. For details, please refer to "Panelists comments and Reply" in each clinical question.

2. We present the level of agreement of each recommendation opinion (level of agreement = the number of panelists agreed / the total number of panelists participating in the survey * 100%).

3. Q: Question; E: Panelists Comments; R: Reply.

| **Clinical question 1: What are the most common symptoms of COVID-19 in children and other criteria that indicated the need of further assessment?** | |
| --- | --- |
| **Consensus recommendation:** | **Level of Agreement** |
| - **The condition of children who have a history of contact with someone infected with COVID-19 should be further assessed. The most common presenting symptoms are fever and cough, with vomiting and diarrhea occurring less frequently. (2C)** | **Consensus** |
| **Panelists Comments and Reply**  **This clinical question has received 11 comments from 8 panelists, our replies to the feedback are as follows：**  Q1: On the problem of “suggestions on imaging and laboratory examination”.   - E1: the word "extend" in the last sentence should be "extent", right? - E2: Change spelling of extend to “extent”. - E3: 1）In rationale, it should be imaging findings or imaging manifestation rather than imaging performance ,because performance is efficacy or ability; It was called ground-glass opacity generally, GGO for short, so I do not know if the ground glass shadow is right because it was expressed as nodular in the study of 15 cases. 2) In evidence summary, we should change multiple leaf segments into multiple lobe segments. - E4: 1) Please change 'extend' to extent，2）. We suggest changing the sentence from “Although there is no significant difference between adults and children in chest imaging characteristics, the extend of the abnormalities are usually less in children” to “Although the chest imaging characteristics of infected adults and children are not significantly different, the extent of abnormalities are usually less severe in children” ? - E5: Spelling: ... the [extent] of the abnormalities ...   R1: We have removed recommendations of imaging and laboratory testing.  Q2: On the problem of “reference format”.   - E1: In the references, the titles of the cited documents are all in lower-case, some in capital letters; in the references, the order of the author's names, some first name in front, some last name in front, which are described in different forms. - E2：first name and last name misplaced in many references with Chinese authors   R2：We have revised our references. Please refer to the full text of the guideline for details.  Q3: On the problem of “whether to emphasize nonproductive dry cough”.   - E1：Should we emphasize in the evidence that cough is nonproductive cough, because this is the clinical differentiation point between viral and bacterial pneumonia.   R3: We have not revised our evidence to emphasize nonproductive cough after discussion.  Q4：On the problem of “negative value in 95% CI of meta-analysis result”.   - E1：Please check the data of Specific symptoms in table 1, because there are six negative values of lower CI limits in table 1。   R4：We do not present tabular data throughout the guideline.  Q5：On the problem of “emphasizing gastrointestinal symptoms”.   - E1：although fever and cough are the main symptoms, do GI symptoms need to highlight   R5：We have revised our recommendation. Refer to the full text of the guideline for details.  Q6：On the problem of “contradictory description of mild and asymptomatic”.   - E1：Notice："......children with COVID-19 are more common have only mild symptoms (93% of children) while about 1/5( 23%)have no symptoms......" This description seems contradictory   R6：We have revised our rationale. Refer to the full text of the guideline for details. | |

| **Clinical question 2: How should children suspected with SARS-CoV-2 infection be managed?** | |
| --- | --- |
| **Consensus recommendation** | **Level of Agreement** |
| - **For children who have a high risk of contact with COVID-19 patients, if no symptoms occur, we suggest they stay at home for observation for a duration of 14 days during which schoolwork continues and attention is given to their psychological well-being. If there are symptoms, their guardians should consult with the healthcare system (depending on arrangements specific to each country) and/or family doctors by telephone call or internet firstly. (2C)** | **Consensus** |
| **Panelists Comments and Replies**  **This clinical question has received 12 comments from 9 panelists, our replies to the feedback are as follows:**  Q1: on the problem of the word“obvious”and“symptoms”   - E1: obvious symptoms, I am not sure whether "obvious" is frequently a clinical term. If using "suspected", is it better? - E2: still about "obvious symptoms", should we use () to explain the specific symptoms? - E3: the last sentence "if there are obvious symptoms", if a child only has fever without cough, just one symptom, should he go to the hospital? Please consider changing as "once /if one suspected symptom happens/occur...". I am not sure whether there is difference between plurality and singular for the word "symptom". - E4: Edit: If no obvious symptoms [are present] ...   R1:We have deleted the word“obvious”in the recommendation. To better explain the word “symptoms”, please refer to our guideline in the clinical question 1. We have changed the word “symptom” to “symptoms”.  Q2: on the problem of “PCR test”   - E1: The sentence "However, it should be noted that depending on the detection reagents and improper clinical sampling in different parts of the respiratory tract, the sensitivity of the nucleic acid test may be as low as 70% [4-5], and some diagnoses may be missed" should be changed as "However, it should be noted that the sensitivity of the nucleic acid test may be as low as 70% [4-5], and some diagnoses may be missed, depending on the detection reagents and improper clinical sampling in different parts of the respiratory tract" - E2: in the rationale,“During the hospital visit the doctors determine whether the child should stay at home under observation, or do a SARS-CoV-2 nucleic acid test as soon as possible”. grammar error. - E3:We suggest 'further evaluation in hospital' to be described in detail. Especially, the when and how for COVID-19 testing must be described. For example, detailed assessment of clinical signs and symptoms by hospital or healthcare provider should be listed for the COVID-19 testing. - E4: In limited testing capacity and its accuracy, making criteria/priority for COVID19 testing is important. Health care providers should have information when and how COVID19 testing (PCR) is to be done for suspected cases. It is because that many people want to be tested, and testing all of the population is impossible. Though I suppose there are limited evidence, I hope the specific criteria including symptoms and signs in the hospital are listed in the guideline.   R2: We have deleted the part of “PCR” and changed the recommendations. Please refer to the clinical question 1 in the guideline to see the details of symptoms and signs.    Q3: on the problem of the word “recommendation”   - E1: The highlighted part "recommend" (rather than "suggest"), does it mean strong recommendation?   R3: We have revised the recommendation.  Q4: “children suspected with SARS-CoV-2” and “children in close contact with COVID-19” are not consistent.   - E1: The question refers to “children suspected with SARS-CoV-2“,whereas the response mentions “children in close contact with COVID-19”. Please keep the terminology consistent.   R4: We haven’t revised this recommendation. This question has been discussed in the expert group and they thought that there was no problem of the expression.  Q5:question of“telemedicine”   - E1: If there is no family physician available for some area, maybe telemedicine is an alternative.   R5: We have revised our recommendation. See the full guideline for details.  Q6: question of change “guardians or family doctors”   - E1: change “guardians or family doctors” into “local or community hospital or clinic in China setting”.   R6: We have revised this phrase. Since our guideline is a global guideline，“China setting” wasn’t added. | |

| **Clinical question 3: Can chest imaging test help diagnose children with COVID-19?** | |
| --- | --- |
| **Consensus recommendation:** | **Level of Agreement** |
| - **CT scan should not be used as routine examination for the diagnosis of children with COVID-19, although it may be helpful in monitoring children who develop severe respiratory symptoms. (2C)** | **R2:79%** |
| **Panelists Comments and Reply**  **This clinical question has received 13 comments from 11 panelists, our replies to the feedback are as follows：**  **Q1:** On the problem of “Recommend using CT in children with COVID-19”.   - E1: I think that CT is sensitive to viral pneumonia, especially in children with COVID-19. It will help us to classify the level of disease earlier. At present, the radiologists prefer low dose CT to find difference in children with suspected pneumonia or the other diseases. So, it will reduce the radiation risk in children. - E2: We suggest not use imaging test as routine examination for children with COVID-19. but for 2019nCoV pneumonia, even if suspected, chest CT examination is better than chest radiography. - E3: children are in the range of 0-18.in older children the CT findings are more typical, even in young children.   **R1:** We have revised our recommendation based on existing evidences. Studies have shown that children are more sensitive to radiation. Under the circumstances that most cases of childhood COVID-19 are mild. Relevant evidence is insufficient whether the use of CT for children have more benefits than harm. Therefore, CT examinations for children should be avoided as much as possible.  **Q2:** On the problem of “Statement of recommendation”.   - E1: grammar issue--please see the highlighted parts--"suggest not using...". For other "against" recommendations (for example, Rec 4 and 5...), we said "recommend against...", in this "against" recommendations, we said "suggest not...", should we keep identical in expression? - E2: Agree with the recommendation. Slight detail: maybe we should specify that the recommendation is related to the diagnosis of COVID-19 (i.e. the use of CT should not be excluded if necessary for other reasons/co-infections/specific symptoms)? Suggested revision could be for example: "Imaging tests should not be used as a routine examination for the diagnosis of COVID-19 in children" - E3: We suggest not using ….? - E4: I generally agree this point, not to use radiology as routine differential diagnosis. Considering the unknown features of COVID-19 in children, and those children with underlying conditions, I suggest to use imaging to make "pneumonia" diagnosis if needed. My point is that, without necessary imaging, some pediatric patients may miss the diagnosis of pneumonia and the dynamic grasp of the disease severity.   **R2:** We have revised our recommendation based on existing evidences. Please refer to recommendation for specific details.  **Q3:** On the problem of “not including X-ray”.   - E1: chest imaging tests include x-ray and CT. however, this recommendation only mentioned CT. Based on the "Reply for comments" documents, I saw there are some comments about X-ray. If we decide we will not mention X-ray in this guideline, should we directly use "chest CT imaging tests"? - E2: Chest imaging includes x-ray and CT scan. The evidence presented is related to CT scan, and there is nothing about x-ray. Further, the question is related to imaging for “diagnosis” of COVID-10, whereas the recommendation does not mention the word “diagnosis". It gives the impression that imaging itself is not recommended. Therefore, the recommendation may be rephrased as: WE suggest that chest imaging by CT scan should not be done for the diagnosis of COVID-19”. - E3: X-ray as routine examination is still necessary.   **R3:** We have revised our recommendation based on existing evidences. Studies have shown that X-ray has high missed diagnosis rate, considering most cases of childhood COVID-19 are mild, the role of X-ray is limited.  **Q4:** On the problem of typos.   - E1: Typos, revised in text.   **R4:** We have revised.  **Q5:** On the problem of “changing the clinical question”   - E1: Do we need to revise the clinical question? For the children, it may not be possible to perform the image examination. For mild cases, we suggest not using image test as routine. For severe cases, imaging test should be recommended, which is helpful for diagnosis and disease assessment.   **R5:** We haven't revised our clinical question after discussion by the panelists.  **Q6:** On the problem of “insufficient literature on adult imaging diagnosis”.   - E1: The number of evidences is increasing. The sentence “Our rapid review included 16 studies with 449 patients, all of them used CT as a diagnostic tool for patients with COVID19.” need to be updated.   **R6:** We have revised our inclusion and exclusion criteria and added supplementary search.  **Q7:** On the problem of “the format of references”.   - E1: The format of references is not uniform, some have magazine names, some have not.   **R7:** We have revised the format of references. . | |

| **Clinical question 4: Should antiviral drugs such as ribavirin, interferon, remdesivir (GS-5734), lopinavir/ritonavir or oseltamivir be used to treat children with COVID-19?** | |
| --- | --- |
| **Consensus recommendation:** | **Level of agreement** |
| - **We recommend against using antiviral drugs for children with COVID-19 outside of a clinical trial. (1C)** | **Consensus** |
| **Panelists Comments and Reply**  **This clinical question has received 7 comments from 4 panelists, our replies to the feedback are as follows**  Q1. On the problem of “reference number”.   - E1：references was showed below, however, it showed 22 references in the above box.   R1：We have revised our references. Refer to the full text of the guideline for details.  Q2: On the problems of “wording of recommendation”.   - E1: We recommend against using antiviral drugs above listed for children with COVID-19. ......   R2: We have revised our recommendation from “We recommend against using antiviral drugs for children with COVID-19. Specific antiviral drugs may be  administered only in the context of clinical trial (1C)” into “We recommend against using antiviral drugs for children with COVID-19 outside of a clinical trial (1C)”.  Q3: On the problems of “rationale wording”   - E1: Antiviral drugs are effective treatment against viruses which can affect the replication cycle of the virus and get them eliminated. This sentence needs to be modified. - E2: It should be chloroquine and hydroxychloroquine instead of chloroquine phosphate. - E3: Suggestions on adding explanation of adverse reactions of these drugs. - E4: Reference forms is still uniform.   R3: We have revised. We have changed the sentence of “Antiviral drugs are effective treatment against viruses which can affect the replication cycle of the virus and get them eliminated” into “Most viral diseases are self-limited illnesses that do not require specific antiviral therapy”, and changed “chloroquine phosphate” into “hydroxychloroquine”. We have added adverse reactions of these drugs in evidence summary from the results of our meta-analysis, and modified the reference form. Refer to the full text of the guideline for details.  Q4: On the problem of “wording of recommendation”.   - E1: Actually, there is no evidence supporting the recommendation “Specific antiviral drugs may be administered only in the context of clinical trial”. Meanwhile, if we use “may be”, the strong recommendation is inappropriate. And 1C should be placed after first sentence.   R4: We have revised our recommendation from “We recommend against using antiviral drugs for children with COVID-19. Specific antiviral drugs may be  administered only in the context of clinical trial (1C)” into “We recommend against using antiviral drugs for children with COVID-19 outside of a clinical trial (1C)”. Refer to the full text of the guideline for details. | |

| **Clinical question 5: Should antibiotic agents be used to treat children with COVID-19?** | |
| --- | --- |
| **Consensus recommendation:** | **Level of Agreement** |
| - **We recommend against the use of antibiotic agents for children with COVID-19 when there is no evidence of bacterial coinfection. (1B)** | **Consensus** |
| **Panelists Comments and Reply:**  **The clinical question received 1 comment from 1 expert. Our replies to the feedback are as follows：**  Q1：On the problem of “The reference 6 need to be confirmed”.  R1：Your suggestion has been accepted. We have made due modifications. Refer to the “evidence summary” part for details. | |

| **Clinical question 6: Should systemic corticosteroids be used to treat children with severe COVID-19?** | |
| --- | --- |
| **Consensus recommendation:** | **Level of Agreement** |
| - **We recommend against using systemic glucocorticoids for children with COVID-19 routinely (1C).** - **We suggest a low dose and a short duration for severe COVID-19 children only when over inflammatory reaction or in the context of clinical trials. (2D)** | **R2:100%** |
|  | **R2:93%** |
| **Panelists Comments and Reply**  **This clinical question has received 13 comments from 11 panelists, our replies to the feedback are as follows：**  Q1: On the problem of “Changes in the wording of the recommendations section”.   - E1: This sentence "We suggest a low dose and a short duration for severe COVID-19 children ....." could be revised to "a low dose and a short duration in using them". otherwise ,we don't know what it is. "only when over inflammatory reaction or in the context of clinical trials." I am not sure whether this expression is correct. Could be revised " only when over inflammatory reaction happen or in the context of clinical trials" ? - E2：Routine use of glucocorticoids is not indicated and not recommended."......when over inflammatory reaction happening or......" - E3: The overall recommendation may be jointly phrased as: We do not recommend the routine use of …………. COVID-19 (1C). However, if the decision to use systemic glucorcticoid is made, only low dose for short duration may be used when there is evidence of excessive inflammatory reaction (2C) or in the context of clinical trials. - E4: Why not describe the second question as “acute respiratory distress syndrome”, instead of “over inflammatory reaction”? How is this defined? There seems to be no such diagnosis. - E5: I would slightly reword the second recommendation (e.g. "We suggest that children with severe COVID-19 can be treated with low-dose short-term systemic glucocorticoid therapy when having over-inflammatory reaction, or in the context of clinical trials.") - E6: I did not find object in the second recommendation. Should we change the recommendation to “We suggest a low dose and a short duration systemic glucocorticoid for severe COVID-19 children only when over inflammatory reaction or in the context of clinical trials. (2D)”? - E7:1). We agree on the emphasis of not using systemic glucocorticoids in routine for children with COVID-19.2). We suggest to change from ' We suggest a low dose and a short duration for severe COVID-19 children only when over inflammatory reaction or in the context of clinical trials' to 'We suggest the use of low dose and short duration systemic glucocorticoids in children with severe COVID-19 only when there is over inflammatory reaction or in the context of clinical trials' - E8: We still suggest a low dose and a short duration of corticosteroids for severe COVID-19 children only in clinical trials.   R1: We haven't revised our recommendation. Refer to the full guidelines for details.  Q2: On the problem of “The duration of use and the dosage of recommended systemic glucocorticoid”   - E1: 1) could we specify the "low dose and short-term" use of systemic glucocorticoid for children in the "rational" part, in order to provide the detailed guidance for clinicians. - E2: If there is currently no accepted definition or standard for small dosage and short courses of treatment. - E3: When and how to use systemic glucocorticoids to prevent cytokine storm is controversial. It is particularly hard to decide in pediatric setting because the lack of trials and studies. We hope we can find the time point of the host to initiate cytokine storm with certain inflammatory markers when more evidence is available.   R2: We haven't revised our recommendation. After careful analysis and consideration of the opinions of the above experts, our research group reviewed the evidence  again. At present, there is no reliable evidence on the dosage and the duration of use of glucocorticoids in children with severe COVID-19. So only the dosage and usage proposed in relevant studies are presented in the evidence summary part.  Q3: On the problem of “There is no clinical evidence that children can use systemic glucocorticoid therapy”.   - E1: Besides, there is no evidence supporting using systemic glucocorticoids in the context of clinical trials.   R3: We haven't revised our recommendation. A case report included one child with severe COVID-19 showed that intravenous methylprednisolone (2 mg/kg, 3 days) combined with interferon, gamma globulin, anti-infective drugs and other symptomatic treatments could improve oxygenation index (including PaO2/FiO2 of 93 to 212 mmHg and oxygenation index of 19 to 7.1) as well as lung function.  Q4: On the problem of “Reference format”.   - E1: The format of references is not uniform.   R4: We have revised our reference format. Refer to the full guidelines for details. | |

| **Clinical question 7: Should intravenous immunoglobulin (IVIG) be used to treat children with severe COVID-19?** | |
| --- | --- |
| **Consensus recommendation:** | **Level of Agreement** |
| - **We recommend against using intravenous immunoglobulin (IVIG) for children with severe COVID-19. (1B)** | **Consensus** |
| **Panelists Comments and Reply**  **This clinical question has received 6 comments from 6 panelists, our replies to the feedback are as follows：**  Q1: On the problem of “the dose of IVIG”.   - E1: We recommend against using intravenous immunoglobulin (IVIG) in for children with severe COVID-19 routinely especially high dose IVIG.   R1: We haven’t revised our recommendation. We haven’t found sufficient evidences to support different doses of IVIG affecting children with COVID-19 differently.  Q2: On the problem of “the format and grammar of the sentences”.   - E1: In addition, a meta-analysis of nine RCTs on infants with sepsis showed IVIG had no reduction in mortality (OR=0.95,95% CI[0.80,1.13]),length of hospital stay (MD=-4.08,95% CI[-6.47,-1.69]), or death or major disability before two years of age (RR=0.98,95% CI[0.88, 1.09]),compared with placebo or no intervention[4]. A few spaces were missing in this paragraph. - E2: Please see the highlighted part--grammar issue. (We recommend against using intravenous immunoglobulin (IVIG) in for children with severe COVID-19)   R2: We have revised the format and the grammar of sentences.  Q3: On the problem of“the data of the outcome of the evidence”.   - E1: In second paragraph of evidence summary, ref 4, the outcome “length of hospital stay (MD=-4.08,95% CI [-6.47, -1.69])” has statistical different. Should we state separately？   R3: The outcome “length of hospital stay (MD=-4.08,95% CI [-6.47, -1.69])” isn’t statistically different.  Q4: On the problem of“high dose effects of IVIG”.   - E1: I basically agree this point. It is not easily ruled out the high dose effects of IVIG in reducing acute vasculitis which may be involved in ARDS development. Needs more study focusing on the specific pathogenesis.   R4: We agree with you.  Q5: On the problem of“whether to mention plasma transfusion therapy”.   - E1: Do we need to mention plasma transfusion therapy?   R5: Our clinical question mainly focuses on IVIG, there is a fundamental difference between IVIG and plasma transfusion therapy. | |

| **Clinical question 8: What are the appropriate means of supportive care for children with severe COVID-19?** | | |
| --- | --- | --- |
| **Consensus recommendation:** |  | |
| **We propose the following forms of supportive care for children with severe COVID-19:**  **1）ensuring sufficient number of adequate health care staff ;**  **2）systematically monitoring and recording vital signs;**  **3）using supportive care of the respiratory and cardiovascular symptoms according to clinical needs;**  **4）providing psychological intervention to children and their family when needed.** | | **Level of Agreement** |
|  |  | **Consensus** |
|  |  | **Consensus** |
|  |  | **Consensus** |
|  |  | **Consensus** |
| **Panelists Comments and Reply**  **This clinical question has received 5 comments from 5 panelists, our reply to the feedback is as follows**  **Q1**: On the problem of“nutrition intervention”   - E1: should we mention the nutrition intervention for children with severe COVID-19?   **R1**: We haven’t added nutrition support in our recommendation after thorough reading of evidence reference and group discussion.  **Q2**: On the problem of “providing psychological intervention to children and their family when needed”   - E1: providing psychological interventions for older children. - E2: psychological support or intervention when needed.   **R2**: We have revised recommendation, but given the differences among children of all ages, we do not provide specific information of interventions.  **Q3**: On the problem of“isolation and quarantined in evidence”  E1: Two words isolation and quarantined used in the last sentence of the evidence. I feel that this article is mainly about patients who are quarantined in hospital, so the latter” quarantined” is wrong, because” quarantined” isolates "normal people" who have not been diagnosed.  **R3**: We have revised evidence. Refer to the “evidence summary” part for details.  **Q4**: On the problem “Rationale should be specific”  E1: Rationale should be specific.  **R4:** We have revised the rationale. Refer to the “rationale” part for details. | | |

| **Clinical question 9: Should mothers with COVID-19 continue to breastfeed their babies?** | |
| --- | --- |
| **Consensus recommendation:** | **Level of Agreement** |
| - **Breastfeeding mothers with COVID-19 should continue to breastfeed if their own health condition allows (1C). Mothers should take appropriate precautions when they contact their babies. (1C)** | **Consensus** |
| **Panelists Comments and Reply**  **This clinical question has received 7 comments from 7 panelists, our replies to the feedback are as follows:**  Q1: On the problem of “consider using "suggest" rather than "recommend" because it is a weak/conditional recommendation”.  R1: We have revised our recommendation as “Breastfeeding mothers with COVID-19 should continue to breastfeed if their own health condition allows (1C). Mothers should take appropriate precautions when they contact their babies. (1C)”.  Q2: On the problem of“emphasis on appropriate precautions in the recommendation”   - E1: It might be better to say this：We recommend mothers should not interrupt breastfeeding under appropriate precautions, including wearing masks and hand hygiene or expressing breast milk as an alternative. Notice：SARS-COV-2 transmission through droplets and closed contact during breastfeeding is possible. - E2: I think the last sentence in the Rationale should appear in the recommendation itself. Mothers are encouraged to take appropriate precautions, including wearing masks, hand hygiene and so on, or expressing breast milk as an alternative. - E3: We suggest to add to, we recommend against mothers interrupting breastfeeding, 'however, strict precautions to avoid transmission must be done.'   R2: We have revised our recommendation as “Breastfeeding mothers with COVID-19 should continue to breastfeed if their own health condition allows (1C). Mothers should take appropriate precautions when they contact their babies. (1C)”.  Q3: On the problem of“evidence details of two included case reports ”   - E1: The evidence stated that there were ten mothers, the first reference had 9 pregnant women, 6 tests were negative, the other three did not say; the second reference reported 1 case was negative.   R3: We have revised our evidence summary, refer to evidence summary for details. There were ten mothers with COVID-19 included, but only seven breast milk samples from seven infected mother has been detected (6 from the first case report and 1 from the second), and all samples were negative.  Q4: On the problem of“in the recommendation, mothers should be specific”   - E1: We recommend against mothers with COVID-19 interrupting breastfeeding？Mothers should be specific.   R4: We have revised our recommendation as“Breastfeeding mothers with COVID-19 should continue to breastfeed if their own health condition allows (1C). Mothers should take appropriate precautions when they contact their babies. (1C)”.  Q5: On the problem of “wait and make sure till virus RNA negative in milk at least for 2 weeks.”   - E1: Needs to be cautions, wait and make sure till virus RNA negative in milk at least for 2 weeks.   R4: We haven’t revised our recommendation and rationale to wait and make sure till virus RNA negative in milk at least for 2 weeks, because by now no evidence indicating nucleic acid of SARS-CoV-2 existing in breast milk. After weighing the pros and cons of breasting and isolation, we insist that the advantages of breastfeeding outweigh the risks related to the possible infection. | |

| **Clinical question 10: How should parents be advised to get information on SARS-CoV-2 infection?** | |
| --- | --- |
| **Consensus recommendation:** | **Level of Agreement** |
| - **We recommend parents to obtain information regularly from academic and official resources rather than social media. (1D)** | **Consensus** |
| **Panelists Comments and Reply**  **This clinical question has received 2 comments from 2 panelists, our replies to the feedback are as follows:**  Q1: On the problem of "social media".  E1：I am still confusion about "social media". many academic journals and authorities have WeChat and/or weibo account (two very popular social medias in China, like WHO, it has twitter account) to issue the news and information. Every morning, I think many people got the updates of COVID-19 from social media account of our authority media. Also, National Health Commission issued the guidelines on its social media account (e.g. WeChat). These reliable social media is very helpful for disseminating the news or information, especially for public.  R1: We have not revised our recommendation, because by now many evidences indicate that most of the social media platforms are full of false information. However, we admit that some information from social media endorsed by the authorities and officials are also reliable.  Q2: On the problem of“the exact expression of ‘COVID-19’and ‘SARS-CoV-2 infection’”.  E1: I think SARS-Cov-2 should be replaced with COVID-19 across the text.  R2: We have not revised the disease name and “SARS-CoV-2 infection” was still adopted after discussion. | |

**Basic Terminology**

| **No** | | **Terms** | **Initial definition** |
| --- | --- | --- | --- |
| 1 | Severe Acute Respiratory Syndrome Coronavirus 2 (SARS-CoV-2) | | A novel coronavirus (CoV) is a new strain of coronavirus that has not been previously identified in humans. The virus that caused the outbreak of respiratory illness first detected in Wuhan, China, is named Severe Acute Respiratory Syndrome Coronavirus 2 (SARS-CoV-2) [1]. This is an enveloped RNA virus belonging to the genus Betacoronavirus [2], with a diameter of 60-140nm . The genetic characteristics of SARS-CoV-2 are different from those of the severe acute respiratory syndrome coronavirus (SARS-CoV) and the Middle East respiratory syndrome coronavirus (MERS-CoV) [2]. The genome of SARS-CoV-2 is suggested to be 88% identical with the genome of two bat-derived SARS-like coronaviruses (bat-SL-CoVZC45 and bat-SL-CoVZXC21) [2]. The official name given by the World Health Organization to the disease caused by SARS-CoV-2 is Coronavirus Disease 2019 (COVID-19)[1].  Ref:  1.WHO. Naming the coronavirus disease (COVID-2019) and the virus that causes it. 2020.https://www.who.int/emergencies/diseases/novel-coronavirus-2019/technical-guidance/naming-the-coronavirus-disease-(covid-2019)-and-the-virus-that-causes-it  2.Lu R, Zhao X, Li J, et al. Genomic characterisation and epidemiology of 2019 novel coronavirus: implications for virus origins and receptor binding. Lancet. 2020;395(10224):565–574. doi:10.1016/S0140-6736(20)30251-8 |
| **Questions and replies：**  **Q1**: More than 85%”is not accurate, please cite the number from the paper published in Lancet.  **R1**: Thanks for your suggestions. We have modified the definition according to your suggestion. | | | |
| 2 | Incubation Period | | Incubation period refers to the interval between exposure to SARS-CoV-2 and the symptom onset. Incubation period of COVID-19 is usually 1 to 14 days, and in most cases between 3 and 7 days [1]. The longest incubation period observed was 24 days in one study [2].  Ref:  1.Li Q, Guan X, Wu P, et al. Early Transmission Dynamics in Wuhan, China, of Novel Coronavirus-Infected Pneumonia [published online ahead of print, 2020 Jan 29]. N Engl J Med. 2020;10.1056/NEJMoa2001316. doi:10.1056/NEJMoa2001316  2.Guan WJ, Ni ZY, Hu Y, et al. Clinical Characteristics of Coronavirus Disease 2019 in China [published online ahead of print, 2020 Feb 28]. N Engl J Med. 2020;10.1056/NEJMoa2002032. doi:10.1056/NEJMoa2002032. |
| **Questions and replies：**  **E1:**   - **Q1:** The last sentence should consider adding "longest" or "at most"... - **Q2:** Change last sentence “is" to “was”   **R1:** Thanks for your suggestions. We rewrite the last sentence.  **E2:**   - **Q1:** “between exposure to COVID-19 and the symptom onset”，”COVID-19”should be changed to “SARS-CoV-2”   **R2:** Thanks for your suggestions. We have modified the definition according to your suggestion. | | | |
| 3 | Route and Mode of Transmission | | The main route of the transmission of SARS-CoV-2 is through respiratory droplets and contact [1]. Transmission may occur through aerosol when exposed to high concentrations of aerosols in a relatively closed environment for a long time [2] and fecal-oral route [3]. Whether transmission can occur through urinary tract, or from mother to infant either in utero [4], perinatally or through breast milk, has not been established yet. Ref:  1.CDC. How COVID-19 Spreads. 2020. https://www.cdc.gov/coronavirus/2019-ncov/about/transmission.html (accessed Feb 16, 2020)  2.New coronavirus pneumonia prevention and control program (7nd ed.) (in Chinese). 2020 [http://www.nhc.gov.cn/yzygj/s7653p/202003/46c9294a7dfe4cef80dc7f5912eb1989/files/ce3e6945832a438eaae415350a8ce964.pdf.](http://www.nhc.gov.cn/yzygj/s7653p/202002/8334a8326dd94d329df351d7da8aefc2/files/b218cfeb1bc54639af227f922bf6b817.pdf.)  3.Xiao F, Tang M, Zheng X, Li C, He J, Hong Z, et al. Evidence for gastrointestinal infection of SARS-CoV-2. medRxiv. 2020:2020.02.17.20023721. doi: 10.1101/2020.02.17.20023721.  4.Chen H, Guo J, Wang C, et al. Clinical characteristics and intrauterine vertical transmission potential of COVID-19 infection in nine pregnant women: a retrospective review of medical records. The Lancet, 2020,DOI:https://doi.org/10.1016/S0140-6736(20)30360-3. |
| **Questions and replies：**  **Q1:** I think oral-faecal route has not been fully proved yet.  **R1:** Thanks for your suggestions. Although the evidence is limited, the general public should be wary of this mode of transmission. | | | |
| 4 | Contact | | Contact is defined as a person who is involved in any of the following [1]:   1. Staying in the same close environment of a COVID-19 patient (including household, classroom, gatherings). 2. Traveling together in close proximity (1 m) with a COVID-19 patient in any kind of conveyance. 3. Direct exposure to body fluids or specimens including aerosols   There are so far no separate criteria for contact specifically for children.  Ref:  1.WHO. Global Surveillance for human infection with coronavirus disease (COVID-19). 27 Feb 2020. https://www.who.int/publications-detail/global-surveillance-for-human-infection-with-novel-coronavirus-(2019-ncov) |
| **Questions and replies：**  **Q1**: Please check whether the literature is true that people who returned to Beijing after >14 days who were initially test negaive, later becane test positive.  **R1**: Thanks for your suggestions. There were rare cases tested positive after >14 days. Therefore we define the time limit as “within 14 days” based on WHO’s recommendation.  **Q2**: Family cluster is more important history than travel in children.  **R2**: Thanks for your suggestions. We have modified it as “Staying in the same close environment of a COVID-19 patient (including **household**, classroom, gatherings)”  **Q3:**.Within approximately 2 meters (6 feet) especially within 1 meter  **R3**: Thanks for your suggestions. We have modified it based on evidence from WHO.  **Q4:** What is the definition of "prolonged period of time" - 30 min (?) or ...  **R4**: Thanks for your suggestions. We are sorry that no evidence have defined "prolonged period of time" so far. | | | |
| 5 | Suspected Case （Person Under Investigation） | | Children who contact with COVID-19 patients have any of the following conditions:   1. any acute viral respiratory illness (fever and/or respiratory symptoms) [1, 2] 2. other symptoms such as gastrointestinal symptoms (vomiting and/or diarrhea) [3] 3. abnormal chest CT with no symptom [4].   Ref:  1.WHO. Global Surveillance for human infection with novelcoronavirus (2019-nCoV)Interim guidance. Jan 31, 2020.<https://www.who.int/publications-detail/global-surveillance-for-human-infection-with-novel-coronavirus-(2019-ncov)>  2.CDC. Evaluating and Reporting Persons Under Investigation (PUI). 2020. <https://www.cdc.gov/coronavirus/2019-ncov/hcp/clinical-criteria.html?CDC_AA_refVal=https%3A%2F%2Fwww.cdc.gov%2Fcoronavirus%2F2019-ncov%2Fclinical-criteria.html>  3.Guan WJ, Ni ZY, Hu Y, et al. Clinical Characteristics of Coronavirus Disease 2019 in China [published online ahead of print, 2020 Feb 28]. N Engl J Med. 2020;10.1056/NEJMoa2002032. doi:10.1056/NEJMoa2002032.  4.Chan JF, Yuan S, Kok KH, et al. A familial cluster of pneumonia associated with the 2019 novel coronavirus indicating person-to-person transmission: a study of a family cluster. Lancet. 2020;395(10223):514–523. doi:10.1016/S0140-6736(20)30154-9 |
| **Questions and replies：**  None | | | |
| 6 | Confirmed Case | | Suspected children with at least one of the following types of pathogenic evidence [1]:   1. positive result for SARS-CoV-2 by Reverse Transcription-Polymerase Chain Reaction (RT-PCR) test for nucleic acid in respiratory or blood or stool samples.   (2) viral gene sequencing showing high homegeneity to SARS-CoV-2 in respiratory or blood or stool samples.  Ref:  1.WHO. Global Surveillance for human infection with novelcoronavirus (2019-nCoV)Interim guidance. Jan 31, 2020.<https://www.who.int/publications-detail/global-surveillance-for-human-infection-with-novel-coronavirus-(2019-ncov)> |
| **Questions and replies：**  None | | | |
| 7 | Asymptomatic Infection | | Children who have been exposed to a confirmed or probable case of COVID-19 [1], and are tested positive for SARS-CoV-2 without manifestations of any clinical symptoms or abnormal chest imaging findings [2].  Ref:  1. WHO. Global Surveillance for human infection with novel coronavirus (2019-nCoV)Interim guidance. Jan 31, 2020.<https://www.who.int/publications-detail/global-surveillance-for-human-infection-with-novel-coronavirus-(2019-ncov)>  2. Shen K, Yang Y, Wang T, et al. Diagnosis, treatment, and prevention of 2019 novel coronavirus infection in children: experts' consensus statement [published online ahead of print, 2020 Feb 7]. World J Pediatr. 2020;10.1007/s12519-020-00343-7. doi:10.1007/s12519-020-00343-7 |
| **Questions and replies：**  None | | | |
| 8 | Mild Case | | Children with a SARS-CoV-2 infection present upper respiratory tract infection (with fever, cough and/or fatigue), without manifestation of pneumonia [1, 2].  Ref:  1.WHO. Clinical management of severe acute respiratory infection when novel coronavirus (nCoV) infection is suspected. Jan 11, 2020. https://www.who.int/internal-publications-detail/clinicalmanagement-of-severe-acute-respiratory-infection-when-novelcoronavirus-(ncov)-infection-is-suspected (accessed Jan 19, 2020)  2.Shen K, Yang Y, Wang T, et al. Diagnosis, treatment, and prevention of 2019 novel coronavirus infection in children: experts' consensus statement [published online ahead of print, 2020 Feb 7]. World J Pediatr. 2020;10.1007/s12519-020-00343-7. doi:10.1007/s12519-020-00343-7 |
| **Questions and replies：**  None | | | |
| 9 | Severe Case | | Children with COVID-19 present the criteria of severe pneumonia (such as breathing faster, dyspnea, respiratory distress, hypoxemia, change of consciousness, or trouble feeding) and critical illness (such as respiratory failure, septic shock or other organ failure requiring intensive care)[1,2].  Ref:  1.WHO. Clinical management of severe acute respiratory infection when novel coronavirus (nCoV) infection is suspected. Jan 11, 2020. https://www.who.int/internal-publications-detail/clinicalmanagement-of-severe-acute-respiratory-infection-when-novelcoronavirus-(ncov)-infection-is-suspected (accessed Jan 19, 2020)  2.Pocket book of hospital care for children: Guidelines for the management of common childhood illnesses . http://www.who.int/maternal_child_adolescent/documents/child_hospital_care/en/]. 2nd ed. Geneva: WHO; 2013. |
| **Questions and replies：**  **Q1**: How about some children previously with chronic disease, such as diabetes, cancer....  **R1**: Thanks for your suggestions. Children with underlying disease may be more likely to become severe cases, but it may not be appropriate to emphasis it in the definition.  **Q2**: ......critical illness (such as ARDS, respiratory failure, sepsis, septic shock or other organ failure requiring intensive care)......  **R2**: Thanks for your suggestions. Sepsis is defined as a life-threatening organ dysfunction caused by dysregulated host response to infection. “Sepsis” and “organ failure caused by infection” may be repetitive when they were both presented in the definition. | | | |
| 10 | Self-Isolation | | If children are asked to self-isolate, they should stay home and avoid going to a public area or attending social gatherings until 14 days after the date of their suspected epidemic contact or exposure to SARS-CoV-2. In addition, their caregivers have to report any symptoms of COVID-19 immediately to their health providers. Appropriate personal protective equipment and hygiene practices should be used when close contact is needed [1,2].  Ref:   1. CDC. Interim Guidance for Preventing the Spread of Coronavirus Disease 2019 (COVID-19) in Homes and Residential Communities.https://www.cdc.gov/coronavirus/2019-ncov/hcp/guidance-prevent-spread.html (accessed Feb 20, 2020) 2. WHO. Home care for patients with suspected novel coronavirus (nCoV) infection presenting with mild symptoms and management of contacts. Feb4, 2020.https://www.who.int/publications-detail/home-care-for-patients-with-suspected-novel-coronavirus-(ncov)-infection-presenting-with-mild-symptoms-and-management-of-contacts (accessed Jan 19, 2020) |
| **Questions and replies：**  None | | | |
| Pathway: Screening and Management of Children with SARS-CoV-2 | | | |
| **Q1:**   - **E1:** Please see the below highlighted part, did it miss “supportive interventions”?   **R1:** Thanks for your suggestion. We have modified it.  **Q2:**   - E1: in community ，“without symptoms” should be evaluated by a community doctor（Family doctor）not parent   **R1:** We changed the recommendation and suggest them to give a telephone call before to see their doctors.  **Q3:**   - **E1:** An asymptomatic person should be given a nucleic acid test if possible.   **R1:** Thank you for your advice. We changed the pathway. | | | |
